# Mitochondria‐Targeted Nanotherapeutics: A Promising Strategy in Modulating Mitochondrial Function, Transfer, and Transplantation

**DOI:** 10.1002/advs.77713

**Published:** 2026-09-27

**Authors:** Yuanyuan Su, Zhiyan Gong, Jiao Mu, Lili Huang, Pengfei Yang, Chenli Zhang, Lingshan Zhao, Liling Yang, Jun Deng, Xiaohui Liao

**Affiliations:** ^1^ Department of Nephrology The Second Affiliated Hospital of Chongqing Medical University Chongqing China; ^2^ Department of Nephrology University‐Town Hospital of Chongqing Medical University Chongqing China; ^3^ Department of Ultrasound The First Affiliated Hospital of Chongqing Medical University Chongqing China; ^4^ Kuanren Laboratory of Translational Lipidology Centre For Lipid Research The Second Affiliated Hospital of Chongqing Medical University Chongqing China; ^5^ Department of Nephrology Second People's Hospital of Yibin Yibin China; ^6^ Department of Nephrology Mianyang Central Hospital Mianyang China; ^7^ Center for Regenerative Medicine Department of Clinical Laboratory The Second Affiliated Hospital of Chongqing Medical University Chongqing China

**Keywords:** mitochondria, mitochondrial transfer, mitochondrial transplantation, nanomaterials, nanotherapeutics

## Abstract

Mitochondria, as organelles with a critical role in maintaining cellular activities, are also vulnerable to dysfunction, which contributes substantially to the pathogenesis of various systemic diseases. Nanomaterials, owing to their tunable physicochemical properties and precise bio‐interfacial capabilities, offer innovative platforms not only for delivering general mitochondria‐targeted therapy but also for enabling—with promising potential—the emerging paradigms of mitochondrial transfer and transplantation. Nevertheless, a dedicated review systematically summarizing progress in this specific subfield is lacking. To fill this knowledge gap, this review provides a structured synthesis that spans from the biological basis of mitochondrial function and transfer to the broad applications of nanomaterials in mitochondria‐targeted therapies, with a dedicated analysis of their roles in mitochondrial targeting, functional modulation, and gene editing. A key topic of this review is the emerging role of nanomaterials in assisting mitochondrial transplantation and transfer. By integrating these insights, this work bridges nanotechnology and mitochondrial medicine, offering a valuable resource for developing organelle‐specific therapeutics.

## Graphical abstract

This review summarizes the pathogenic role of mitochondria in diseases and highlights mitochondrial transfer and transplantation as emerging therapeutic strategies. It systematically discusses how nanomaterials are engineered to facilitate these processes, and critically examines the current challenges and future perspectives for their clinical translation.

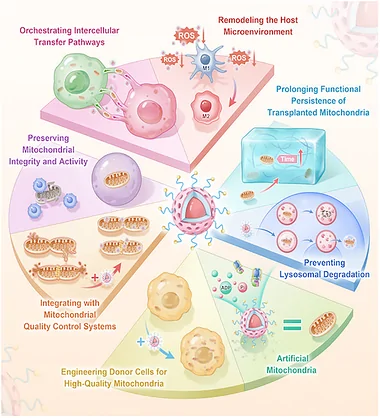

## 1 Introduction

Mitochondria are pivotal organelles that regulate diverse cellular processes, including energy supply [1], cell death [2, 3], signal transduction [4, 5], and redox homeostasis [6]. Consequently, mitochondrial dysfunction underlies a wide spectrum of diseases, including cardiovascular [7, 8], neurodegenerative [9], and metabolic disorders [10, 11], as well as cancer [12] and autoimmune diseases [13, 14, 15]. The central role of mitochondria in cellular homeostasis makes them a promising therapeutic target.

Current mitochondria‐targeted therapeutic strategies can be broadly categorized into three classes. The first therapeutic approach focuses on modulating mitochondrial functions [16], such as regulating energy production, influencing mitochondrial reactive oxygen species (mtROS) production, and quality control. The second strategy involves replacing the damaged mitochondria with exogenous healthy ones [17], which can compensate for functional deficits or induce mitophagy to restore cellular homeostasis. More recently, a third class of mitochondrial therapeutics has emerged that leverages the natural process of intercellular mitochondrial transfer for endogenous supplementation [18]. This approach avoids the risk of immunogenicity inherent to the use of foreign mitochondria. These three types of strategies have considerable therapeutic potential for a broad range of applications. However, these therapies share a common limitation: the inability to precisely target mitochondria, leading to low efficacy and off‐target effects. Moreover, mitochondrial transplantation is impeded by rapid inactivation of mitochondria post‐isolation, while intercellular mitochondrial transfer suffers from inherently low efficiency [19, 20].

Nanomaterials, with their finely tunable size (typically 1–100 nm), shape, and surface chemistry, offer a versatile platform to address these challenges. They enable precise mitochondrial targeting through strategies that mimic or conjugate with targeting ligands [21]. The integration of multiple targeting strategies further facilitates the use of a hierarchical approach, ensuring specificity across tissue, cellular, and organelle levels. Beyond targeting, nanomaterials can orchestrate critical mitochondrial processes, including energy metabolism [22], mtROS dynamics [23], and biogenesis [24]. During mitochondrial transplantation applications, nanomaterials function as protective carriers that preserve organellar integrity [25]. For intercellular mitochondrial transfer, nanomaterials improve efficiency by promoting the formation of intercellular connections [26, 27] and enhancing biogenesis in donor cells [28]. In addition to facilitating delivery, nanomaterials can remodel the local microenvironment [29] and provide sustained therapeutic release [30]. Recently, nanomaterials have been explored as direct mitochondrial mimics to rescue cellular function [31, 32, 33, 34]. Nevertheless, despite the transformative potential of nanomaterials in mitochondrial medicine, a systematic synthesis of their application, particularly in advancing mitochondrial transplantation and intercellular transfer, is currently lacking.

Here, this review provides a comprehensive analysis of the application of nanomaterials in mitochondria‐targeted therapy, with a novel focus on two emerging strategies, namely mitochondrial transplantation and mitochondrial transfer, to bridge the current knowledge gap. First, we summarize the foundational biology of mitochondria and the mechanisms regulating their transfer. Subsequently, we critically evaluate current nanomaterial‐based strategies for mitochondrial targeting, functional modulation, and mitochondrial DNA (mtDNA) gene editing. Finally, we highlight the transformative potential of nanomaterials in facilitating mitochondrial transplantation and transfer. By synthesizing these advances, we contend that nanomaterial‐assisted mitochondrial transplantation and transfer represent a new direction for treating multiple diseases. Through a critical discussion of design principles and major challenges, this review seeks to inspire innovative solutions that will accelerate the clinical adoption of mitochondria‐targeted therapies.

## 2 The Structure and Function of Mitochondria

As essential organelles in eukaryotic cells, mitochondria possess a highly organized structure and perform multiple critical functions, as illustrated in Figure 1.

**FIGURE 1:**
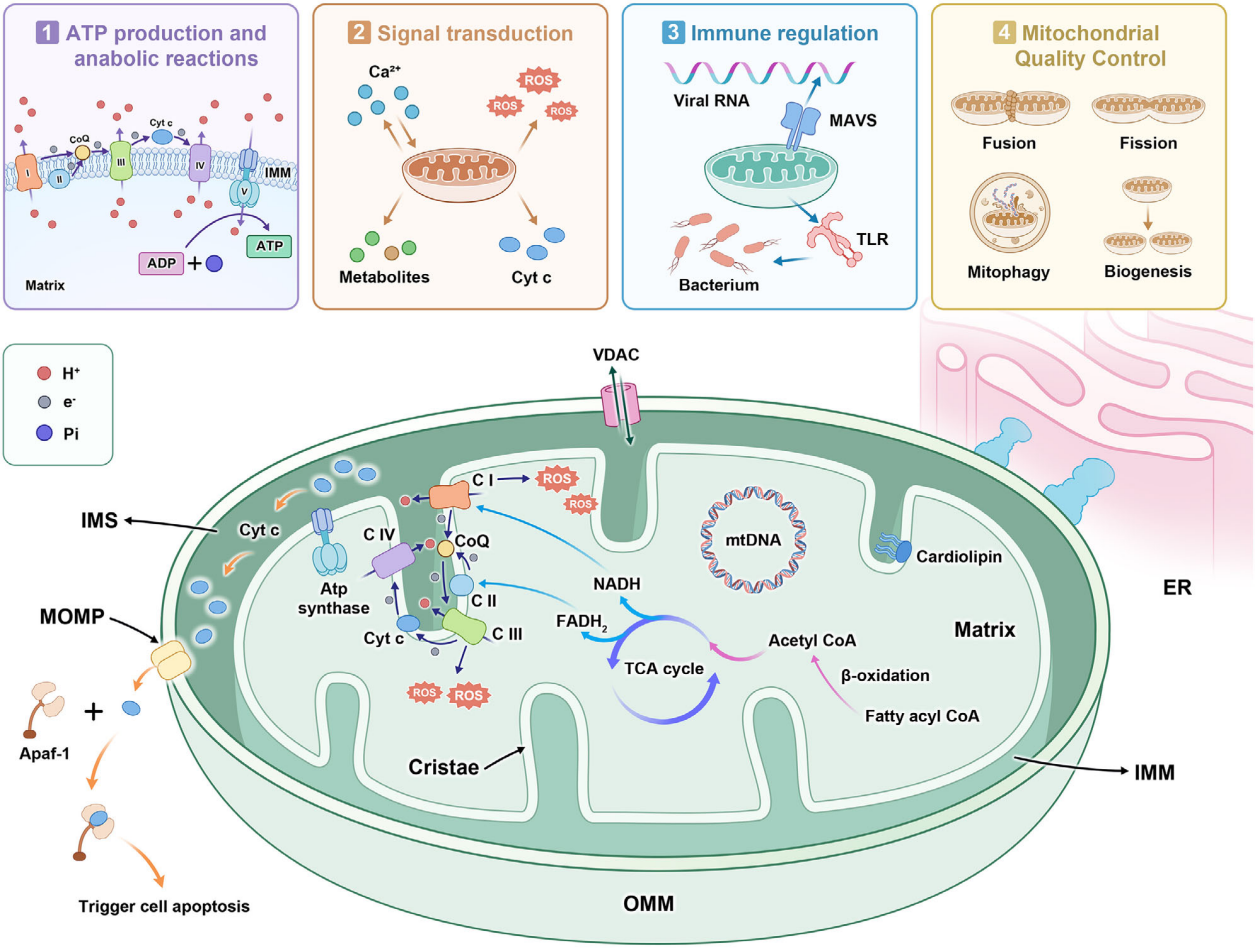
The structure and function of mitochondria. Mitochondria consist of four compartments: outer mitochondrial membrane (OMM), intermembrane space (IMS), inner mitochondrial membrane (IMM), and matrix. They serve key functions in ATP production and anabolic reactions, signal transduction, immune regulation, and mitochondrial quality control.

### 2.1 Basic Structure of Mitochondria

According to the leading theory, mitochondria evolved from an alpha‐proteobacterium that was engulfed by an archaeal host nearly two billion years ago [35]. This endosymbiotic event marked the rise of eukaryotes, and the bacterium's circular DNA was retained, forming the mitochondrial genome. As highly dynamic organelles, mitochondria exhibit considerable heterogeneity in morphology, size, and network architecture across different cell types.

Internally, the double membrane of mitochondria partitions the organelle into four distinct compartments: the outer mitochondrial membrane (OMM), the inner mitochondrial membrane (IMM), the intermembrane space (IMS), which is encompassed by the OMM and IMM, and the matrix, which is inside the IMM.

The matrix houses the enzymatic machinery for the tricarboxylic acid (TCA) cycle and fatty acid β‐oxidation. It also contains mtDNA—the organelle's own genetic material—and its associated transcription/translation system. mtDNA is a small, approximately 16 kbp, circular mitochondrial genome. While the nuclear genome encodes the majority of mitochondrial proteins, the mtDNA specifically encodes 13 core subunits of the electron transport chain (ETC) complexes. The mtDNA is organized into nucleoprotein structures with mitochondrial transcription factor A (TFAM) serving as its primary packaging and binding protein [36].

The IMM forms cristae by folding itself to increase surface area for achieving maximal function [37]. The IMM, which is the main energy production site for aerobic respiration, contains the protein complexes of oxidative phosphorylation [OXPHOS; complex I, II, III, IV, and the enzyme ATP synthase (complex V)] and associated metabolic transporters [38]. Cardiolipin is predominantly localized to the IMM, where it acts as a signaling hub by engaging in non‐covalent interactions with diverse proteins [39].

The IMS houses key proapoptotic proteins such as cytochrome *c* (Cyt *c*, bound to the IMM) and apoptosis‐inducing factor. The OMM houses pore‐forming proteins that act as channels for material transport and platforms for interaction with other organelles [40].

Mitochondria and the endoplasmic reticulum (ER) are pivotal organelles in eukaryotes, and the direct interfaces between them constitute essential hubs for cellular organization and regulation. The contact sites between the OMM and ER are termed mitochondria‐ER contact sites (MERCS), which are also known as mitochondria‐associated ER membranes. These junctions play an important role in calcium (Ca^2+^) signaling, conduction, and lipid transfer, as well as in cellular homeostasis [41, 42].

### 2.2 Function of Mitochondria

#### 2.2.1 ATP Production and Anabolic Reactions

As the cellular powerhouse, the primary role of mitochondria is ATP generation to provide the essential energy for cell survival. OXPHOS is the central energy production pathway in most mammalian cells. This process involves the ETC, which comprises protein complexes I–IV, and the mobile electron carriers ubiquinone (coenzyme Q) and Cyt *c*, which ultimately drives ATP synthesis through ATP synthase. During ATP generation, excessive reactive oxygen species (ROS) are produced by protein complexes I and III as a side product [43]. Beyond their canonical role in oxidative catabolism, where molecules lose electrons, mitochondria also facilitate reductive anabolism by adding electrons to acceptor molecules to drive biosynthetic reactions [6]. This anabolic capacity allows mitochondria to support cell proliferation.

#### 2.2.2 Signal Transduction

Mitochondria play a key role in regulating intracellular Ca^2^^+^ levels. They take up and release Ca^2^^+^ through specialized transporters, most notably the mitochondrial calcium uniporter, a highly selective ion channel.

Mitochondrial ROS participates in cell signal transmission. Oxidative stress occurs when cellular antioxidant defenses are subdued by excessive levels of ROS. This leads to detrimental oxidation of cellular components, including lipids, proteins, nuclear DNA, and mtDNA. The resulting genomic instability and accumulation of damaged mtDNA ultimately trigger cell death through apoptosis or necroptosis and contribute to mitochondrial destruction [44, 45]. ROS induce Ca^2+^ influx into mitochondria and affect calcium homeostasis, leading to mitochondrial dysfunction [46].

Mitochondria release metabolites that serve as essential substrates and cofactors for epigenetic modifications of DNA and histones in the nucleus [47]. In addition to this intracellular signaling role, certain mitochondrial intermediates, such as succinate, can function as extracellular signaling messengers [48].

Mitochondria are critical regulators of intrinsic apoptosis, pyroptosis, and ferroptosis [3]. During intrinsic apoptosis, proapoptotic proteins Bax/Bak oligomerize in the OMM, triggering mitochondrial outer membrane permeabilization (MOMP) and releasing Cyt *c* into the cytosol [49]. Cyt *c* then binds to apoptotic protease activating factor 1 (Apaf‐1) and pro‐caspase‐9 to initiate the caspase cascade and cell death [2]. Mitochondrial damage is also a critical driver of pyroptosis [50] and plays a pivotal early‐stage role in deciding between apoptosis and nucleotide‐binding oligomerization domain (NOD)‐like receptor family pyrin domain containing 3 (NLRP3) inflammasome‐mediated pyroptosis [51]. Alterations in mitochondrial morphology and function, such as metabolic rewiring and diminished ATP production, are hallmark features of ferroptosis and may actively contribute to its execution. MERCS also contribute to promoting ferroptosis by acting as key hubs for phospholipid peroxidation [52].

#### 2.2.3 Immune Regulation

Mitochondria regulate immune responses by integrating signaling and metabolic pathways. In response to bacterial or viral infection, mitochondria undergo dynamic morphological changes and metabolic reprogramming to modulate cell apoptosis. Additionally, during bacterial infection, mitochondria contribute to Toll‐like receptor (TLR)‐mediated bactericidal activity through ROS generation, and during viral infection, they initiate an interferon response through the mitochondrial antiviral signaling protein (MAVS) signaling pathway [53].

Mitochondria regulate signaling pathways downstream of key innate immune receptors, including retinoic acid inducible protein I (RIG‐I)‐like receptors, NOD‐like receptors, and TLRs [54, 55, 56, 57]. Mitochondrial metabolic reprogramming is crucial for immune cell activation, differentiation, and survival, thereby fulfilling both signaling and effector functions [58, 59, 60, 61, 62].

Numerous studies have shown that mitochondrial components can be recognized as damage‐associated molecular patterns (DAMPs) by immune molecules [63, 64]. Both oxidized mtDNA release and excessive ROS levels serve as potent triggers for the nucleation of the NLRP3 inflammasome. Mitochondria also provide a structural platform for inflammasome assembly through molecules such as cardiolipin, mitofusin 2 (Mfn2), and MAVS, thereby facilitating the maturation and release of inflammatory cytokines [65].

#### 2.2.4 Mitochondria Quality Control

Mitochondria are highly dynamic networks. Cells adapt to increased metabolic demand and stress through a mitochondrial quality control (MQC) system that preserves mitochondrial integrity. MQC is a critical homeostatic system maintained by four coordinated processes: fusion, fission, mitophagy, and biogenesis. Mitochondrial fusion, mediated by mitofusins (Mfn1/2) and optic atrophy 1 (OPA1) [66], enables content exchange between organelles, complementing damaged components and averting their premature destruction. Conversely, fission, promoted by dynamin‐related protein 1 (DRP1) and its adapters [67], generates smaller mitochondria to facilitate their distribution and transport. For irreparable damage, mitophagy selectively eliminates defective mitochondria through PINK1/Parkin‐dependent or PINK1/Parkin ‐independent pathways, preventing further cellular harm [68]. To replenish the network, peroxisome proliferator‐activated receptor‐γ coactivator‐1α (PGC‐1α) activates mitochondrial biogenesis by upregulating nuclear respiratory factors (NRF1/2), which promote the expression of mtDNA‐ and OXPHOS‐related genes [69]. Collectively, these dynamic processes ensure functional integrity by renovating the mitochondrial network, relocating organelles to meet energy demands, and enabling adaptive responses to stress.

## 3 Mitochondrial Dysfunction in Various Diseases

Given the diverse functions of mitochondria, their dysfunction has been established as a pivotal factor in the pathogenesis of several diseases (Figure 2).

**FIGURE 2:**
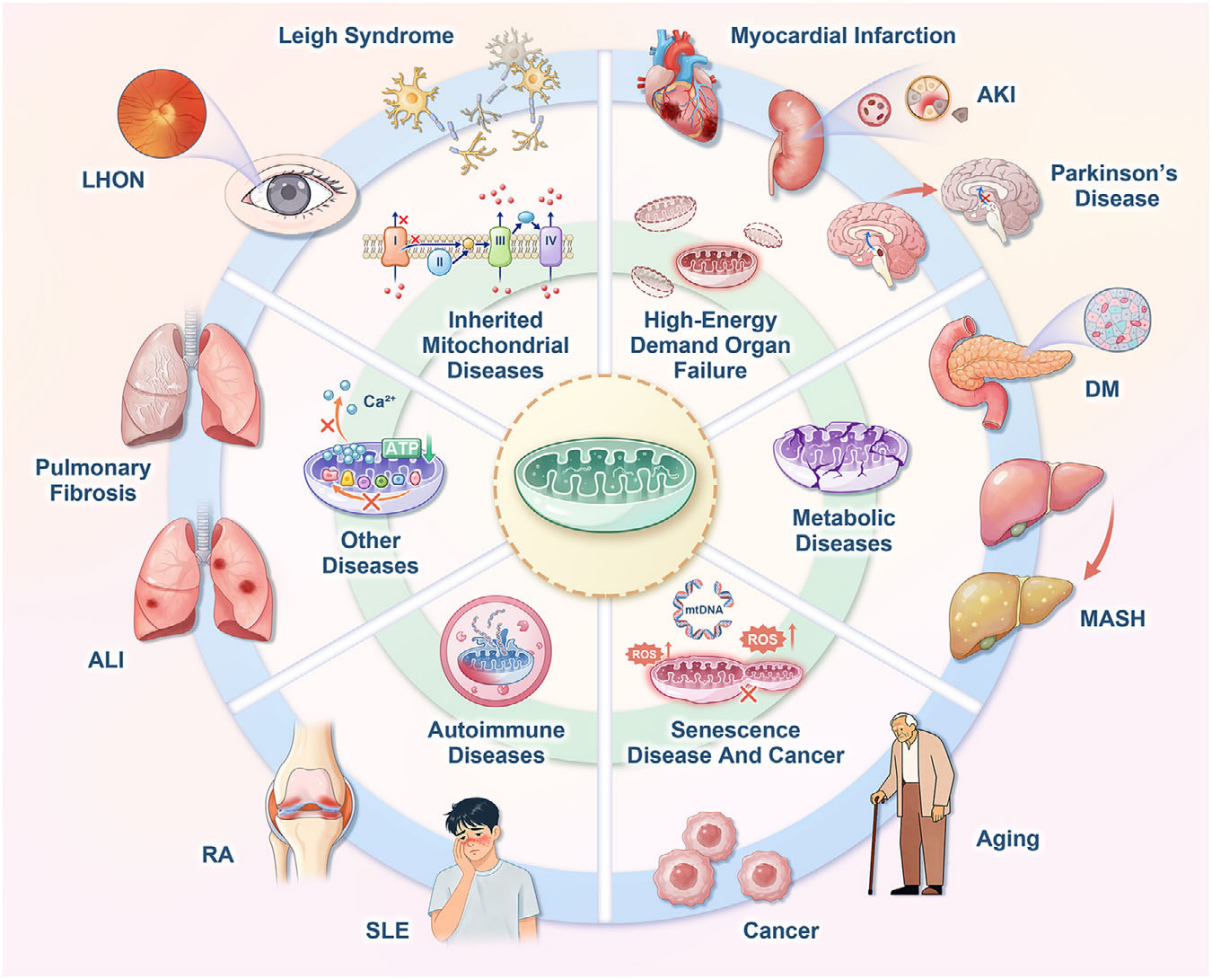
Mitochondrial dysfunction in various diseases. Mitochondrial dysfunction contributes to a broad spectrum of human diseases, broadly classified into inherited mitochondrial diseases (e.g., Leigh syndrome and Leber hereditary optic neuropathy (LHON)), high‐energy demand organ diseases (e.g., myocardial infarction, Parkinson's disease, and acute kidney injury (AKI)), metabolic diseases (e.g., diabetes mellitus (DM) and metabolic dysfunction‐associated steatohepatitis (MASH)), senescence‐related diseases and cancer (e.g., aging and various malignancies), and autoimmune diseases (e.g., systemic lupus erythematosus (SLE) and rheumatoid arthritis (RA)), as well as other conditions such as pulmonary fibrosis and acute lung injury (ALI).

Mitochondrial diseases can be either congenital or acquired in nature. Inherited mitochondrial disorders are typically caused by genetic defects. Acquired mitochondrial dysfunction manifests across a broad range of conditions. A reduction in mitochondrial copy number is particularly detrimental to tissues with high energy demands, often leading to severe clinical consequences. In addition, other manifestations of mitochondrial dysfunction, including metabolic reprogramming, ROS imbalance, and aberrant dynamics, have been increasingly recognized as critical contributors to the pathogenesis of diverse disorders, such as metabolic diseases, autoimmune conditions, cancer, and ageing.

### 3.1 Inherited Mitochondrial Diseases

Both nuclear DNA and mtDNA encode mitochondrial contents; consequently, mutations in either of them might induce mitochondrial dysfunction.

The manifestation of an inherited mitochondrial disease depends on the existence of specific mtDNA mutations and the ratio of mutant mtDNA to wild‐type mtDNA within a cell. Leber's hereditary optic neuropathy (LHON) is a representative inherited mtDNA disease and often presents with a homoplasmic or high‐level heteroplasmic mutation. LHON is caused by complex I dysfunction in ocular mitochondria, resulting in a decline in energy production and an increase in ROS levels, contributing to central vision degeneration [70]. Leigh syndrome (LS) is a severe infant‐onset neurometabolic disorder. It is genetically heterogeneous, with pathogenic variants in nuclear‐encoded genes, such as *NDUFS4*, and several mtDNA genes, which collectively disrupt ETC function [71].

### 3.2 Mitochondrial Dysfunction in High‐Energy‐Demand Tissues

Tissues with high energy demands, such as the heart, kidney, and brain, are densely packed with mitochondria and are therefore particularly vulnerable to mitochondrial dysfunction [72, 73, 74].

In the cardiovascular system, mitochondrial dysfunction manifests as disordered substrate metabolism, defective OXPHOS, and impaired energy shuttling in heart failure. It also accelerates cardiomyopathy and atherogenesis through defective mitophagy and elevated ROS, and contributes to ischemia/ reperfusion injury via Ca^2+^ overload and mitochondrial permeability transition pore (mtPTP) opening [7, 8, 75]. In the kidney, the high metabolic demand of proximal tubular cells renders them particularly susceptible to mitochondrial dysfunction under hypoxia, toxic, or metabolic disturbances, contributing to both acute kidney injury (AKI) and chronic kidney disease [76]. In the brain, mitochondrial dysfunction is implicated in neurodegenerative disorders and ischemic stroke, with oxidative stress, Ca^2+^ overload, and impaired MQC playing key pathogenic roles [77, 78, 79].

### 3.3 Mitochondrial Dysfunction in Metabolic Diseases

Mitochondrial dysfunction drives diabetes by promoting insulin resistance in skeletal muscle through reduced fatty acid oxidation, lipid accumulation, and excessive ROS production, while also impairing pancreatic β‐cells via insufficient ATP generation and oxidative stress that ultimately lead to β‐cells apoptosis [80, 81, 82]. Additionally, oxidative stress and altered mitochondrial membrane potential (MMP) and bioenergetics due to mitochondrial dysfunction contribute to podocyte damage, leading to kidney injury in a hyperglycemic environment [11, 83].

Dysfunctional mitochondrial remodeling has been observed in both visceral and subcutaneous adipose tissues of individuals with obesity [84]. White adipose tissue is critical for systemic lipid flux, but mitochondrial dysfunction compromises its lipid storage capacity, causing free fatty acids and cytokines to spill into circulation for uptake by the liver and skeletal muscle [85].

In metabolic dysfunction‐associated steatohepatitis (MASH), hepatocytes harbor mitochondria with significant structural and functional deficits. These impairments lead to reduced OXPHOS efficiency and elevated ROS production [86]. The resulting oxidative stress damages hepatocytes and, crucially, activates quiescent hepatic stellate cells (HSCs) [87], thereby initiating a profibrotic response that promotes fibrosis progression.

### 3.4 Mitochondrial Dysfunction in Senescence, Disease, and Cancer

Recent studies have established mitochondrial dysfunction as a key feature of cellular senescence, playing a causal role in both induction and maintenance of the senescent state [88, 89]. During aging, these dysfunctional changes are characterized by impaired metabolic capacity, elevated ROS production, and accumulation of mtDNA mutations [90].

Beyond the well‐established Warburg effect [91], mitochondria also influence tumor development through ROS regulation, metabolic reprogramming, and dynamic quality control, playing a complex role in tumor initiation, progression, and metastasis [92].

### 3.5 Mitochondrial Dysfunction and Autoimmune Diseases

Mitochondrial dysfunction drives autoimmune and inflammatory diseases through mtDNA release, impaired mitophagy, oxidative stress, and metabolic dysregulation. In systemic lupus erythematosus (SLE), mtDNA release and defective mitophagy activate inflammatory responses [15]. In rheumatoid arthritis (RA), mtDNA mutations, sustained oxidative stress, and dysregulated cellular energy metabolism contribute to pathogenesis [14].

In inflammatory bowel disease (IBD), the high energy demand of intestinal epithelial cells makes them particularly vulnerable to deficits in ATP production. The damaged mtDNA, following its release, acts as an inflammatory trigger, with its levels correlating with disease severity. mtROS impairs the intestinal barrier and recruits immune cells, while both mtROS and microbial signals activate the NLRP3 inflammasome to sustain chronic inflammation [93].

### 3.6 Role of Mitochondrial Dysfunction in Other Diseases

Mitochondria influence the pathogenesis of acute lung injury (ALI) through energy metabolism dysfunction, dysregulation of calcium homeostasis, oxidative stress, and induction of immune cell pyroptosis [94]. Mitochondrial dysfunction in alveolar epithelial cells (AECs), stemming from a reduced functional mitochondrial load and impaired mitophagy, plays a crucial role in the pathogenesis of pulmonary fibrosis (PF) [95, 96]. Mitochondrial dysfunction is also critically involved in PF progression by influencing the differentiation of fibroblasts into myofibroblasts [97].

## 4 Intercellular Mitochondrial Transfer

Mitochondria themselves can act as critical intercellular mediators. The mitochondrial membrane has a rich content of cholesterol and is structurally analogous to eukaryotic membranes, which facilitate its internalization by recipient cells. In 2006, Spees et al. first demonstrated that, following co‐culture with mesenchymal stem cells (MSCs) or human skin fibroblasts, human lung cancer cells lacking mtDNA acquired mitochondria and mtDNA. This transfer caused loss of uridine and pyruvate auxotrophy, restored respiratory function and OXPHOS, and enabled resumption of exponential cell growth [98]. Since then, mitochondrial transfer is increasingly recognized as a critical mechanism in several physiological and pathological contexts such as ischemic stroke, cancer progression, and allograft rejection.

### 4.1 Multifaceted Roles of Mitochondrial Transfer in Health and Disease

#### 4.1.1 Mitochondrial Transfer in Physiological and Pathological Conditions

Thymic epithelial cells rely on exogenous mitochondrial transfer from early thymic progenitor cells for performing normal physiological functions [99]. Intercellular mitochondrial transfer also serves as a crucial pathway for immunometabolic interactions between adipocytes and macrophages, thereby regulating metabolic homeostasis. This vital crosstalk mechanism is impaired in obesity [100]. Notably, while the basal rate of mitochondrial transfer is generally low under physiological conditions, it is markedly augmented by pathological stimuli [101].

Mitochondrial transfer is mostly initiated by cellular distress signals. Babenko et al. reported that mitochondrial transfer from MSCs to astrocytes is amplified under ischemic conditions with high ROS levels [102], which can activate the PI3K/AKT signaling pathway—a recognized regulator of this transfer process. Acidic stress promotes mitochondrial transfer among human corneal limbal epithelial cells [103]. Mild hypothermia (33°C) can enhance mitochondrial transfer from astrocytes to oxygen–glucose deprivation/reoxygenation‐injured neurons [104]. Some toxic chemical compounds induce mitochondrial transfer as a compensatory response. Cobalt chloride, despite inducing injury in pheochromocytoma 12 (PC12) cells, can enhance the efficiency of mitochondrial transfer from MSCs to PC12 cells [105]. Similarly, commonly used engineered nanomaterials such as titanium dioxide nanoparticles (NPs) and multi‐walled carbon nanotubes can also promote this compensatory mitochondrial transfer [106].

These stress‐induced mechanisms of mitochondrial transfer play significant roles across diverse disease contexts. For instance, in cardiovascular diseases, mitochondrial migration is observed among different cells, including cardiomyocytes, endothelial cells (ECs), and vascular smooth muscle cells, under physiological conditions [107, 108, 109]. The functional outcome of this transfer is contingent upon multiple factors, including the intrinsic state of mitochondria and donor cell properties.

In MASH, dysfunctional mitochondria transferred to hepatocytes increase the population of lipid droplet‐bound mitochondria, disrupting lipid metabolism and promoting lipid accumulation, mtROS overproduction, and apoptosis [110].

Impaired cells can also take up functional mitochondria to improve their biological capacity and rescue their function. In diabetic nephropathy, high glucose stimuli enhance the uptake of MSC‐derived mitochondria by macrophages. This transfer promotes M2 macrophage polarization and activates PGC‐1α‐mediated mitochondrial biogenesis [111]. Osteocytes support the normal architecture of transcortical vessels (TCVs) by transferring mitochondria to TCV ECs, thereby reversing endothelial dysfunction [112].

#### 4.1.2 Transmitophagy Through Mitochondrial Transfer

Excessive mitochondrial stress can disrupt lysosomal function and impair mitophagy. To overcome this impairment, stressed cells can expel mitochondria for uptake and degradation by neighboring cells, a process termed transmitophagy. The released mitochondria can also act as a distress signal [113].

In the optic nerve head, impaired mitochondria in retinal ganglion cells are transferred to adjacent astrocytes for lysosomal degradation [113]. Similarly, in adipose tissue, adrenergically stressed adipocytes secrete damaged mitochondrial components within EVs. These extracellular mitochondria are subsequently cleared by resident macrophages, a process essential for sustaining thermogenesis [114].

#### 4.1.3 Mitochondrial Transfer as a Double‐Edged Sword in Cancer

Horizontal mitochondrial transfer was discovered in various types of cancers such as lung cancer, pancreatic cancer, breast cancer, and glioblastoma [115]. Mitochondrial transfer within the tumor microenvironment (TME) reveals a complex network of intercellular communication. This transfer occurs not only among tumor cells themselves but also between tumor cells and various cells in that environment, including immune cells [116, 117], stromal cells such as fibroblasts [118], ECs [119], homologous normal cells [120], and platelets [121].

Cancer cells, which often harbor high levels of mtDNA damage and mitochondrial dysfunction, can benefit from acquiring functional mitochondria. The nervous system supports tumor metabolism and metastatic dissemination through intercellular mitochondrial transfer from neurons to cancer cells, a process that enhances the mitochondrial function of the recipients and consequently increases their metastatic capacity [122].

Cancer cells can also function as donor cells for mitochondrial transfer to modify their immediate environment for facilitating proliferation. Cancer cells transfer mitochondria with mutated mtDNA to tumor‐infiltrating lymphocytes (TILs), replacing their native mitochondria. Unlike native TIL mitochondria, which are susceptible to ROS‐induced mitophagy, the tumor‐derived counterparts are resistant to it. Consequently, T cells populated with tumor‐derived mitochondria exhibit diminished cytotoxic responses, ultimately promoting tumor immune escape [117]. Co‐culture studies have validated intercellular mitochondrial transfer from tumor cells into fibroblasts in several types of carcinomas. Cancer cell‐derived mitochondria can reprogram fibroblasts into cancer‐associated fibroblasts, and recipient fibroblasts exhibit hallmarks of metabolic activation. These reprogrammed fibroblasts can enhance cancer cell proliferation and migration, which represents a direct cell contact‐mediated mechanism for modulating the TME and promoting cancer cell invasiveness [118, 123, 124].

Cancer cells can also gain chemotherapy resistance through mitochondrial transfer. Chemotherapy‐resistant triple‐negative breast cancer (TNBC) cells donate mitochondria harboring the mutant gene *mtND4* to chemotherapy‐sensitive TNBC cells through extracellular vesicles (EVs), thereby enhancing chemoresistance [125].

Another noteworthy observation is that mitochondrial transfer from bone marrow‐derived mesenchymal stromal cells (BMSCs) to CD8^+^ T cells enhances the metabolic fitness of the latter cell population. Subsequently, this bioenergetic boost mediates the superior antitumor response of CD8^+^ T cells and prolongs their survival in animal tumor models [126].

### 4.2 Intercellular mtDNA Transfer

mtDNA, as one of the mitochondrial components, can be released into the extracellular space. Extracellular mtDNA exists in different forms, including intact free mtDNA, fragmented mtDNA, and vesicle‐encapsulated mtDNA. During myocardial ischemia/reperfusion (I/R) injury, small EVs derived from cardiomyocytes carry mtDNA into cardiac fibroblasts. This internalized mtDNA subsequently activates the STING signaling pathway, which promotes fibroblast activation and proliferation, thereby initiating the process of myocardial fibrosis [127].

Highly metastatic tumor cells can transfer mtDNA to their low‐metastatic counterparts and stromal cells through EVs. This horizontal transfer of mtDNA enhances the metastatic potential of recipient tumor cells while concurrently reprogramming stromal cells within the TME [128]. In prostate cancer, polymorphonuclear myeloid‐derived suppressor cells internalize EV‐packaged mtDNA released from senescent tumor cells. This internalization triggers cGAS‐STING‐mediated type I IFN and NF‐κB signaling pathways, augmenting the immunosuppressive capacity of these suppressor cells through the induction of arginase 1 (Arg1), nitric oxide synthase 2 (Nos2), and programmed death‐ligand 1 (PD‐L1), thereby inhibiting T cells and promoting treatment resistance [129].

### 4.3 Mechanisms of Intercellular Mitochondrial Transfer

While mitochondrial length undergoes dynamic changes due to fission and fusion, the organelles generally range from 0.5 to 1.0 µm, considerably larger than most biomolecules. For the intercellular delivery of such large cargo, mitochondria are transferred between donor and recipient cells through diverse modes (Figure 3), which can be broadly divided into contact‐dependent and contact‐independent modes [130]. Notably, cells can also employ compound strategies to transfer mitochondria.

**FIGURE 3:**
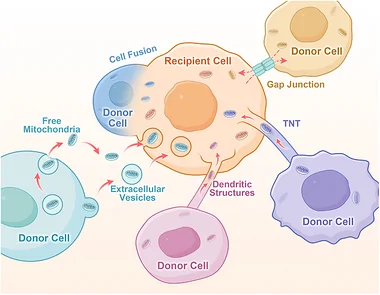
Cells employ a diverse array of mechanisms to transfer mitochondria, forming a dynamic rescue and communication network. Tunneling nanotubes are transient channels that allow the direct delivery of functional mitochondria. Gap junctions are composed of connexins, and they facilitate short‐range intercellular interactions. Extracellular vesicles package and shuttle mitochondrial fragments or entire mitochondria to recipient cells. Free mitochondria can be released into the extracellular space and subsequently taken up by neighboring cells. Dendritic structures extend from the cell surface to transfer mitochondria. Lastly, cell fusion enables the complete merging of cytoplasmic contents, including mitochondrial populations.

#### 4.3.1 Tunnelling Nanotubes

Tunneling nanotubes (TNTs) are submicrometer‐thin membrane channels that form direct connections between cells. TNTs range from 50 to 1500 nm in diameter and 5 to 200 µm in length, with thickness up to 700 nm [131]. As structurally versatile intercellular bridges, TNTs facilitate the trafficking of various organelles and biomolecules—including vesicles, proteins, RNA, and viruses.

Notably, TNTs are recognized as one of the most prominent routes for intercellular mitochondrial transfer across diverse cell types such as neuronal, immune, cancerous, and stem cells. This process is critically governed by mitochondrial Rho‐GTPase1 (Miro1), a calcium‐sensitive adaptor protein that orchestrates the transport of mitochondria through TNTs by forming the mitochondrial motor‐adaptor complex [132].

#### 4.3.2 Gap Junctions

Gap junctions (GJs) are intercellular channels composed of connexins, with a pore diameter of 1.5–2 nm [107]. GJs facilitate short‐range intercellular interactions, permitting the transfer of materials smaller than those transferred by TNTs. Traditionally, GJs have been considered to have a very small diameter, which is not suitable for the direct transfer of mitochondria. Nevertheless, a previous study reported the transfer of intact mitochondria enclosed within double‐membrane vesicles. These structures, known as connexosomes or annular gap junctions, form as a result of gap junction internalization [133].

Beyond their direct role as intercellular conduits, connexins—particularly connexin 43 (Cx43)—have also been implicated in mitochondrial transfer. This occurs through signaling pathways that are independent of gap junction channel function, such as the regulation of TNT formation [134, 135] and stability [133].

Together, these findings establish GJs and their molecular components as important facilitators of intercellular mitochondrial trafficking, acting through both connexosome‐mediated and TNT‐regulatory mechanisms.

#### 4.3.3 Extracellular Vesicles

EVs are lipid bilayer‐enclosed structures released by cells. EVs range from 30 nm to several micrometers in diameter and encapsulate cellular components such as mitochondria, RNA, DNA, and proteins [136]. Based on size, EVs are categorized as small EVs (<200 nm) or large EVs (>200 nm). Based on their biogenesis, EVs can be further divided into distinct subpopulations: (1) exosomes, which originate from the endosomal system, (2) microvesicles (MVs), which bud directly from the plasma membrane and are typically larger than exosomes, and (3) apoptotic bodies and migrasomes. Among these, intact mitochondria have been found to be enclosed within larger EV subtypes, whereas exosomes are physically constrained by their small size (30‐150 nm).

While direct cell‐cell contact enables the proximal transfer of mitochondria, EVs provide a mechanism for the long‐range dissemination of mitochondria. EVs also inherit intrinsic and natural targeting characteristics from their originating cells [137] and can even transport cargoes across impermeable biological barriers, such as the blood–brain barrier (BBB) [138]. However, the efficacy of EV‐mediated mitochondrial transfer is constrained by EV heterogeneity and limited circulation time.

#### 4.3.4 Free Mitochondria

Cells can expel free mitochondria into the extracellular space and circulation, a process often associated with cellular stress and quality control. This route offers a direct but inefficient means of mitochondrial release. These naked mitochondria are susceptible to rapid clearance by debris‐engulfing mechanisms and lack inherent targeting specificity, limiting their ability to reach and be taken up by recipient cells. In contrast to the protective encapsulation afforded by TNTs, EVs, or cell fusion, free mitochondria remain fully exposed to the extracellular surroundings, which not only accelerates their elimination but also compromises their structural integrity and functional activity. As a result, free mitochondrial transfer is generally considered a low‐efficiency pathway compared with other more sophisticated intercellular transfer mechanisms.

#### 4.3.5 Other Mitochondrial Transfer Mechanisms

Additional routes of intercellular mitochondrial transfer have been documented. For instance, high‐resolution confocal imaging has revealed mitochondrial transfer within the osteocytic dendritic network, where healthy osteocytes donate mitochondria to adjacent metabolically stressed cells, thereby restoring their bioenergetic capacity. This process relies on MERCS and is predominantly mediated by Mfn2, a GTPase that tethers the ER to mitochondria [139].

Cell fusion represents another route for mitochondrial exchange. Upon fusion of plasma membranes, the cytoplasmic contents of two or more cells, including mitochondria, become mixed and shared. This process can occur spontaneously or be induced by stimuli such as viral infection or chemical exposure [140]. However, the physiological relevance of cell fusion as a dedicated mitochondrial transfer mechanism remains debated, because cell fusion also gives rise to multinucleated cells such as osteoclasts and giant cells.

The key features, advantages and limitations of each mitochondrial transfer pathway are summarized in Table 1 for the direct comparison.

**TABLE 1:** Different pathways of mitochondrial transfer.

Transfer pathway	Definition	Mechanism of transfer	Advantages	Limitations
Tunneling nanotubes	Membrane channels forming direct physical connections between cells	Direct cell–cell contact via actine‐based transport coordinated mainly by Miro1	Direct, cargo protection	Requires cell–cell contact
Gap junctions	Intercellular channels composed of connexins	Connexosome‐mediated internalization mediated by connexins (particularly Cx43)	Direct, cargo protection	Requires cell–cell contact, short‐range communication
Extracellular vesicles	Lipid bilayer‐enclosed structures released by cells	Vesicle‐mediated delivery and membrane fusion	Long‐range delivery, intrinsic targeting	Heterogeneity, short circulation time
Free mitochondria	Naked mitochondria expelled into extracellular space and circulation	Passive release	Direct	Rapid clearance
Dendritic structure	Intercellular connections through the end‐feet of dendritic structures	Transfer through dendritic processes via Mfn2‐dependent MERCS	Direct, cargo protection	Limited generalizability
Cell fusion	Merging of plasma membranes of two or more cells to form a shared cytoplasmic space	Complete membrane fusion and cytoplasmic mixing	Direct, cargo protection	Physiological relevance debated

### 4.4 Comparison of Mitochondrial Supplementary Therapy

Given the critical roles of mitochondria in cellular homeostasis, some research has explored their therapeutic potential through mitochondrial transfer and transplantation. While intercellular mitochondrial transfer occurs endogenously and spontaneously, therapeutic mitochondrial transplantation is an artificial process that involves the isolation of exogenous mitochondria, followed by in vitro preservation and subsequent administration to target tissues. In this context, exogenous mitochondria are expected to provide an immediate and controllable energy supply.

However, the therapeutic application of mitochondrial transplantation is hindered by challenges at each step of the process. Despite the complexity associated with mitochondrial isolation, isolated mitochondria are vulnerable to rapid inactivation during preservation [141], especially at Ca^2+^ concentrations exceeding 1.3 mM in cell culture media [142]. Delivery routes for mitochondrial transplantation are primarily categorized into local and systemic administrations, each posing distinct barriers. For systemic administration, intravenous injection is the most common route, followed by intraperitoneal injection, intranasal instillation, and oral administration [143]. Intranasal delivery, a non‐invasive method, enables the transport of mitochondria into the central nervous system by effectively bypassing the BBB [144, 145]. However, a major challenge for systemic administration is the lack of targeting ability. Local application, for example, intracoronary mitochondrial transplantation in the heart, enables specific cardiac targeting and significantly increases coronary blood flow. When administered following temporary regional ischemia, this mitochondrial transplantation approach remarkably improves myocardial function and tissue perfusion after I/R injury [146]. Similarly, intramyocardial, intradiscal, and intra‐articular administrations enable rapid distribution and high local concentrations of mitochondria at the target site [147, 148]. Nevertheless, local application usually requires invasive operation, which might cause mitochondrial degradation and local tissue damage. Moreover, large‐volume local administration could potentially lead to adverse outcomes. For instance, intramyocardial injection of a large number of mitochondria has been reported to cause arrhythmias [149].

In addition, exogenous mitochondria can elicit immunogenicity problems. As DAMPs, free extracellular mitochondria can induce inflammation. High levels of macrophage activation at the injection sites have been reported, suggesting the induction of local inflammatory responses [150]. Extracellular mitochondria in circulation can activate neutrophils, exerting immunostimulatory effects [151]. In the related condition of organ allograft, extracellular mitochondria present in donor blood can trigger immune activation following organ transplantation, which may lead to early allograft dysfunction. These mitochondria activate vascular ECs, which represent the first barrier in grafts. They promote the production of inflammatory factors and chemokines and induce dendritic cells to upregulate co‐stimulatory molecules. Animal studies have confirmed that the infusion of isolated mitochondria into donor hearts markedly increases transplant rejection [152].

In contrast, the mitochondrial transfer approach takes advantage of the inherent capacity of cells to spontaneously donate and receive mitochondria, thereby avoiding the need for complex mitochondria isolation and preservation procedures. Nevertheless, this approach is limited by low transfer efficiency, as well as by the difficulty of finding suitable carrier cells that can fulfill the multifunctional requirements of targeted migration and recognition of pathological cells, robust mitochondrial content, spontaneous willingness of cells to initiate mitochondrial donation, and efficient and controlled transfer. Furthermore, the long‐term functional status and potential consequences of transferred mitochondria within recipient cells remain poorly understood, which further constrains its clinical translation.

The key distinctions between mitochondrial transfer and transplantation in terms of implementation process, energy supply, immunogenicity, and major limitations are summarized in Table 2.

**TABLE 2:** Comparison of mitochondrial transfer and mitochondrial transplantation.

Feature	Mitochondrial transfer	Mitochondrial transplantation
Implementation process	Endogenous, spontaneous process leveraging the inherent capacity of cells to donate and receive mitochondria	Artificial multi‐step process requiring isolation, preservation, and administration; administration can be local or systemic
Energy supply	Context‐dependent	Immediate and controllable energy supply
Immunogenicity	Endogenous process with low immunogenicity	High immunogenicity due to extracellular mitochondria acting as DAMPs
Challenges & limitations	Low transfer efficiency; difficulty in identifying suitable carrier cells; unknown long‐term functional consequences in recipient cells	Rapid inactivation of isolated mitochondria during preservation; poor targeting ability of systemic administration; tissue damage and arrhythmias caused by invasive local procedures

### 4.5 Mitochondria Internalization

TNTs, GJs, dendritic structures, and cell fusion—mitochondrial transfer processes that depend on the formation of direct intercellular connections—enable mitochondria to directly enter recipient cells and facilitate the proximal transfer of intercellular mitochondria. As a cell‐contactless mechanism, EVs are internalized by recipient cells mainly through endocytosis or direct fusion with the plasma membrane [153], while recipient cells usually take up free mitochondria through endocytosis, particularly macropinocytosis.

Al Amir Dache et al. traced mitochondria internalized in recipient cells and revealed the dual fate of free mitochondria after internalization. While the majority of free mitochondria co‐localized with endosomal and lysosomal markers, indicating degradation through the endo‐lysosomal pathway, a subset (approximately 9% of the internalized mitochondria) was found to escape this compartment. This escape is mediated by endosomal membrane rupture. Ultimately, these free mitochondria can integrate into the mitochondrial network of the recipient cell [154]. Cowan et al. performed artificial mitochondrial transplantation and found that most internalized exogenous mitochondria (approximately 45% of the total mitochondria) could escape from endosomes and lysosomal chambers and finally integrate with the endogenous mitochondrial network [155].

The fates of transferred and transplanted mitochondria are determined by multiple factors such as the entry pathway, functional status of the mitochondria, intended physiological consequences, conditions of both donor and recipient cells, and disease context. Nevertheless, regardless of whether exogenous mitochondria are functional or nonfunctional, they can activate mitophagy, enhancing mitochondrial biogenesis [156].

Mechanistically, following internalization, exogenous mitochondria induce mitophagy through the canonical PINK1‐Parkin signaling pathway, leading to their eventual degradation within autophagosomes. Some studies suggest that exogenous mitochondria induce mitophagy to clear impaired mitochondria, thereby promoting cell function [157]. In contrast, some other studies observed that exogenous mitochondria reduced mitophagy and apoptosis of recipient cells [158]. The inconsistency in these results might be due to different time periods of detecting mitophagy markers. According to some researchers, exogenous mitochondria induce mitophagy at an early stage [157]. Another study indicated that the improvement in mitochondrial function following supplementation of exogenous mitochondria is the consequence of a repair mechanism, which leads to reduced mitophagy [158].

## 5 Advanced Mitochondrial Targeting and Delivery Strategies

### 5.1 Mitochondrial‐Targeting Paradigms

Because the mitochondrion is an internal cell organelle, its precise targeting is a crucial issue that needs to be addressed to achieve successful mitochondrial therapy. A common design strategy for mitochondria‐targeting molecules is to leverage key mitochondrial properties such as negative membrane potential and lipid composition.

Triphenylphosphonium (TPP), a cationic compound with hydrophobic properties, can accumulate in the mitochondria through electrostatic interactions. The cell‐permeant fluorescent dye Rhodamine 123 accumulates extensively within mitochondria, a process influenced by its cationic and lipophilic properties, which facilitates both cellular uptake and mitochondrial incorporation [159]. Dequalinium chloride (DQA) is a bicationic compound comprising two symmetrical molecules. It can self‐assemble in aqueous solutions to form cationic vesicles known as DQAsomes. These positively charged DQAsomes subsequently accumulate in mitochondria through electrostatic interactions [160].

Szeto‐Schiller (SS) peptides intrinsically enter cells and localize to the IMM through electrostatic and hydrophobic interactions with cardiolipin [159]. It exerts antioxidant effects and has low toxicity toward mitochondria. Elamipretide (SS‐31) is the most commonly used SS peptide. The interaction between SS‐31 and cardiolipin facilitates mitochondrial cristae stabilization, mitigates oxidative stress, and enhances ATP production, thereby ameliorating mitochondrial dysfunction [161]. Mitochondria import macromolecules, such as proteins, through a dedicated protein import machinery that recognizes the mitochondrial targeting signal (MTS). Although this system ensures high specificity for endogenous proteins, its application for delivering exogenous therapeutics is limited because MTS‐linked cargoes cannot efficiently cross the plasma membrane. To acquire both cellular uptake and mitochondrial targeting capabilities, mitochondria‐penetrating peptides (MPPs) were developed by integrating cationic and lipophilic amino acids [162].

### 5.2 Nanomaterial‐Enabled Mitochondrial Targeting

An ideal mitochondrial targeting paradigm should have precise targeting ability, stability, low toxicity, and controlled release features. Sometimes, the desired mitochondrial targeting approach would need to penetrate specific barriers such as the BBB.

A notable limitation of TPP is its tendency to act as a mitochondrial uncoupler at concentrations exceeding 10 µM. By increasing proton leakage across the IMM, it dissipates the proton gradient and can potentially lead to cytotoxic effects [163]. The administration of peptides such as SS peptides has concerns related to their stability and bioavailability. In circulation, peptides are susceptible to proteolytic degradation, structural instability, and interactions with blood components, thereby leading to a short plasma half‐life and reduced bioavailability [164].

Nanomaterials could serve as a platform to overcome the abovementioned shortcomings, while also enabling mitochondrial targeting through alternative mechanisms (Figure 4).

**FIGURE 4:**
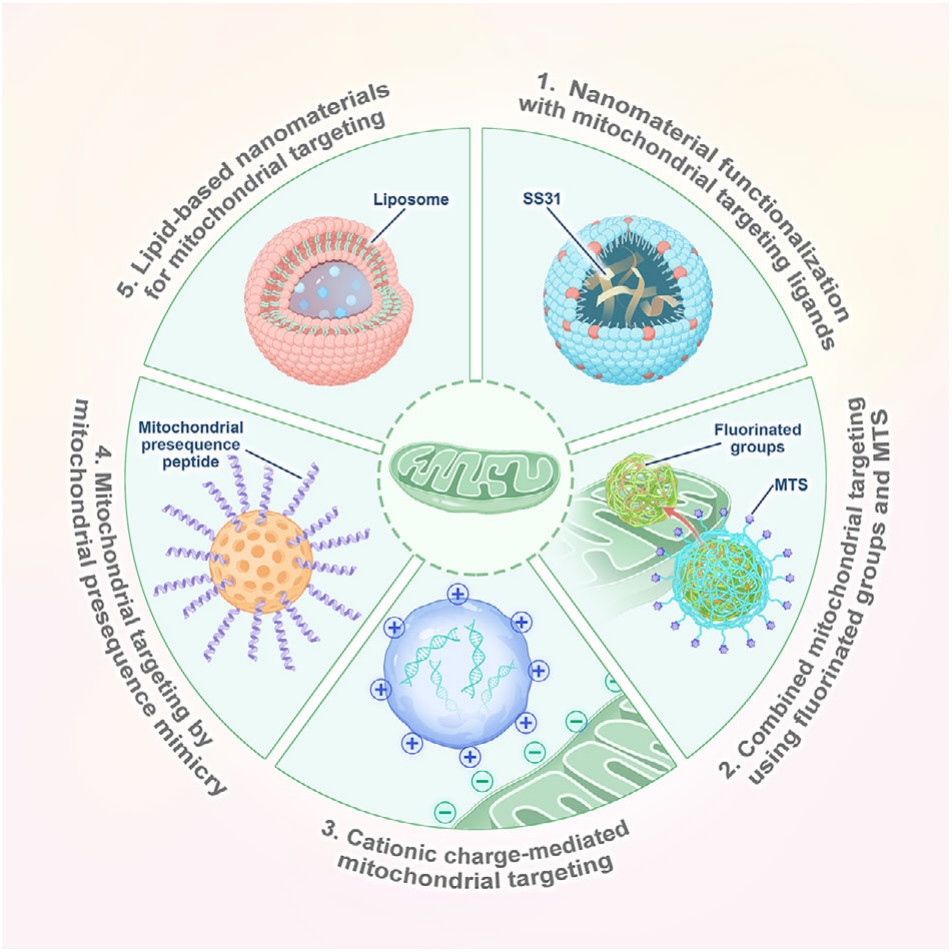
Schematic illustration of nanomaterial‐based mitochondrial targeting strategies. Five representative approaches are depicted, all of which exploit or mimic conventional mitochondrial targeting ligands. These include ligand‐conjugated nanocarriers, dual targeting with mitochondrial targeting signal (MTS) and fluorinated groups, cationic charge‐mediated targeting, surface modification with mitochondrial presequence‐mimicking peptides, and liposome‐based mitochondrial delivery.

Nanomaterials can be used for mitochondrial targeting by conjugating with mitochondrial targeting components such as SS‐31 [161, 165] and SS‐05 [166]. For instance, to resolve the issue of susceptibility of the short peptide SS‐31 to proteolytic degradation, a viable strategy is encapsulation within pH‐responsive chitosan‐based nanopolyplexes [167]. The conjugation of NPs with TPP for mitochondrial targeting could decrease the toxicity of TPP and integrate capabilities for enhanced biocompatibility, bioavailability, and controlled drug release [168].

Under pathological conditions that compromise MMP, the targeting efficiency mediated by cationic hydrophobic groups is substantially reduced because of their high MMP dependence. To achieve membrane potential‐independent mitochondrial gene delivery, Wang et al. developed fluorinated lipid NPs (F‐LNPs). They incorporated fluorinated groups into ionizable lipid tails, which facilitates direct translocation of cargoes into the mitochondrial matrix—bypassing the OMM and IMS. This action is mediated by the interaction of the fluorinated group with the mitochondrial‐specific lipid cardiolipin. To further enhance selectivity, an MTS was added to the optimal formulation, resulting in F‐MLNPs [169].

NPs can achieve mitochondrial targeting through their positive surface charge that favors accumulation in mitochondria [170]. Wang et al. found that by mitigating electrostatic repulsion, isolated mitochondria can achieve a 1.5‐fold higher delivery efficiency [171]. A mitochondria‐regulated information processing (MIP) nanosystem mimics the alkaline amino acid domain of the natural mitochondrial precursor protein to achieve precise cell‐to‐organelle targeting. Due to this sequence homology, it can directly cross the TOM and TIM22 machinery into the mitochondrial matrix [172].

Liposomes have a phospholipid bilayer structure, which is often interlaced with cholesterol and encloses an aqueous interior; this structural design creates distinct hydrophilic and hydrophobic compartments. Jiang et al. reported a mitochondria‐targeted liposome (L‐G2R‐DA) engineered for tumor‐specific phototherapy. The core component is a dendritic lipopeptide engineered with peripheral arginine residues to mimic mitochondrial import signals, leveraging multivalency for superior mitochondrial targeting. A stearoyl group enhances lipophilicity for membrane integration, while an acid‐labile 2,3‐dimethylmaleic acid (DA) moiety enables TME‐responsive charge reversal, shielding cationic charges in circulation and activating mitochondrial affinity specifically within tumor cells. This lipopeptide was co‐assembled with soy phosphatidylcholine, cholesterol, polyethylene glycol‐distearoyl phosphatidylethanolamine (DSPE‐PEG_2000_), and the photosensitizer indocyanine green to form L‐G2R‐DA liposomes. The resulting formulation showed a 3.7‐fold improvement in mitochondrial accumulation compared to TPP‐based liposomes and achieved complete tumor eradication in a 4T1 breast cancer mouse model through potent photodynamic therapy/photothermal therapy effects [173].

The MITO‐Porter system is a liposomal nanocarrier, and its external surface is functionalized with octaarginine (R8) cell‐penetrating peptides, which mediate cellular uptake through macropinocytosis. The carrier leverages electrostatic interactions and membrane fusion to deliver its cargo directly into mitochondria [174].

Nanomaterials can enable a hierarchical targeting strategy from the tissue and cellular levels down to the organelles. Liposomes conjugated with TPP and linked with rabies virus glycoprotein can penetrate the BBB to achieve mitochondrial targeting. By coating the red blood cell (RBC) membrane with this compound, the biocompatibility and long‐term circulation ability of antioxidants encapsulated in nanostructured lipid carriers were improved [175]. Sun et al. developed a dual‐targeting NP system by functionalizing RBC membrane‐coated NPs with two synthetic ligands: C3‐peptide for targeting neural cell adhesion molecule to facilitate central neuron uptake and SS‐31 peptide for mitochondrial localization. This strategy facilitated high intra‐mitochondrial concentrations of cargoes in the central neuron [176].

## 6 Nanomaterial‐Based Strategies for Modulating Mitochondrial Function

Nanotechnology offers a robust platform for enhancing the pharmacokinetic and biodistribution profiles of drug molecules. By using the nanoformulation approach, key physicochemical properties of drugs, including solubility, lipophilicity, and circulatory half‐life, can be optimized, and immunogenicity can be minimized. Additionally, the co‐delivery of multiple drugs through nanocarriers can yield synergistic effects, significantly enhancing therapeutic potency. Because of these benefits, several studies are examining the application of nanomaterials to regulate mitochondrial function.

### 6.1 Nanomaterial‐Mediated Regulation of Mitochondrial Energy Production

Because the primary function of mitochondria is ATP generation, some studies employed nanomaterials to modify this functionality, thereby altering disease outcomes, particularly in cancer treatment. Compared to other cancer cells, TNBC cells mainly rely on OXPHOS for energy supply and require a high amount of copper for ATP generation. Copper‐depleting NPs can reduce OXPHOS by depriving copper in mitochondria of TNBC cells and switch the cell metabolic mode to glycolysis. The decrease in ATP generation, together with compromised MMP and elevated oxidative stress, leads to the apoptosis of TNBC cells [170]. Leng et al. developed a nanomaterial composed of poly (lactic‐co‐glycolic acid) (PLGA) and hyaluronic acid (HA) (termed NBP@PLGA‐HA), which was loaded with the OXPHOS inhibitor nebivolol hydrochloride and the glycolysis inhibitor 3‐bromopyruvate and decorated with CD44 receptor‐targeted HA for specific tumor targeting. This NP can bidirectionally block energy supply of tumor cells [177].

### 6.2 Controlling Oxidative Stress Using Nanomaterials

Nanomaterials conjugated with antioxidant drugs can regulate oxidative stress. Zheng et al. engineered a mitochondrial‐targeted nanogel, TPP‐SAT, through in situ polymerization of TPP, superoxide dismutase (SOD), and catalase (CAT). This system targets mitochondria and utilizes an enzymatic cascade to convert detrimental ROS (•O_2‐_ and H_2_O_2_) into harmless O_2_. Thus, it alleviates hypoxia and oxidative stress, thereby restoring the M1/M2 macrophage equilibrium and reducing inflammation and immune dysfunction. The disruption of this “ROS‐inflammation‐hypoxia” vicious cycle by TPP‐SAT inhibits alveolar bone loss and accelerates periodontal regeneration, showing clinical potential to treat periodontitis and related chronic inflammatory conditions [178]. Gong et al. engineered a mitochondrial‐targeted delivery system by loading the SS‐31 peptide and the antioxidant compound selenium into Treg‐derived exosomes. This system alleviates intestinal inflammation by scavenging excessive mtROS and concurrently inhibiting the integrated cell death pathway PANoptosis with precise targeting, offering a promising therapeutic strategy for IBD [179].

Some nanomaterials can themselves function as antioxidant agents. Nanozymes, which are synthetic structures engineered from materials such as metals, metal oxides, or carbon‐based materials, exhibit enzymatic catalytic activities for various chemical reactions. For instance, cerium dioxide (CeO_2_) can function as a multi‐enzyme mimetic, exhibiting both CAT and SOD activities. Gao et al. developed a nanozyme TBP@CeO_2_ by combining CeO_2_ with black phosphorus and TPP; this nanozyme shows multienzyme activities with mitochondrial targeting. It effectively restored mitochondrial complex II activity and mitigated acetaminophen‐induced liver injury [180]. He et al. developed a nanomaterial using the zeolitic imidazolate framework‐8 carrying CeO_2_ and the Rho‐associated kinase (ROCK) inhibitor Y‐27632, which can repair damaged mtDNA. The NLRP3 inflammasome pathway is suppressed following the internalization of this nanomaterial. This suppression reduces the mtPTP‐mediated leakage of oxidatively damaged mtDNA. Consequently, these events promote the polarization of macrophages toward the M2 phenotype and increase the production of anti‐inflammatory factors [181]. Macrophage membrane‐coated PLGA NPs were developed by co‐encapsulating MnO_2_ to reprogram mitochondrial metabolism in M1 macrophages. These NPs scavenge mtROS and catalyze H_2_O_2_ decomposition to release oxygen and S‐methylisothiourea to inhibit nitric oxide (NO) production. Functionalized with TPP, these NPs selectively deliver their cargo to mitochondria of M1 macrophages, thereby effectively normalizing their aberrant metabolic state [182].

The nanozyme MnFe_2_O_4_ initiates a therapeutic cascade by decomposing H_2_O_2_ to sustainably generate O_2_ and scavenge ROS. It ameliorates the pathological microenvironment by scavenging ROS and generating O_2_. Subsequently, it inhibits apoptosis and activates Ca^2+^‐AMPK‐mTOR mitophagy, thereby restoring mitochondrial function. The resultant metabolic reprogramming from glycolysis to oxidative phosphorylation promotes M2 macrophage polarization, leading to the inhibition of osteoclastogenesis and improvement of osseointegration in RA [183].

The synergistic effects of bimetallic nanozymes enhance catalytic performance by augmenting enzymatic activity and optimizing electronic structure. Their activity and selectivity are precisely adjustable through metal ratio, nanostructure, and surface modification, aided by superior stability that facilitates customization of nanozyme design. Because inflammatory mediators and ROS play a pivotal role in modulating pain perception, Cheng et al. developed a mitochondrial‐targeted TPP‐Au‐Ru nanozyme, which demonstrated high affinity for the mitochondrial matrix, effectively scavenging ROS and reducing inflammatory mediators in vitro and in vivo. It also suppressed the activation of the MAPK and NF‐κB signaling pathways and protected mitochondrial integrity. Thus, the TPP‐Au‐Ru nanozyme alleviated nociceptive symptoms for up to 36 h with minimal biocompatibility issues [184].

Metal‐phenolic networks (MPNs) are constructed through the coordination of metal ions with the catechol or gallol group. These antioxidant nanozymes combine the inherent radical scavenging capability and high biocompatibility of natural phenols with the distinctive electronic, magnetic, and optical properties of the constituent metal ions. By using this strategy, Huang et al. fabricated MPNs termed as Cur‐Cu by coordinating copper (II) (Cu^2+^)—a common cofactor in native SOD—with the well‐studied natural antioxidant curcumin (Cur). This design integrates the ROS scavenging properties of both metal ions and polyphenols. Based on its robust ability to chelate redox‐active metal ions such as Cu^2+^, curcumin prevents •OH generation through Fenton‐like reactions, thus mitigating oxidative damage. Following covalent modification with polyethylene glycol (PEG) to form Cur‐Cu@PEG, the NPs effectively protected HK‐2 cells from ROS‐triggered oxidative damage. The kidney protective effects of NPs were validated in vivo in both cisplatin‐induced AKI and rhabdomyolysis‐induced AKI mouse models [23].

### 6.3 Nanomaterial‐Based Strategies for MQC

Nanomaterials in combination with certain drugs can perform MQC. Sun et al. designed a nanosystem that can deliver olaparib to brain neuronal mitochondria. This strategy inhibited injury‐induced poly (ADP‐ribose) polymerase overactivation, improved mitochondrial function, and prevented neuronal cell death, demonstrating efficacy in traumatic brain injury models [176].

The BaTCG nanosystem, a TPP‐functionalized piezoelectric drug delivery platform, orchestrates a multi‐mechanism therapeutic cascade. Following ultrasound stimulation, it generates a piezoelectric current that promotes mitophagy, thereby reducing ROS production. Combined with the release of encapsulated glucagon‐like peptide‐1 receptor agonists, this dual action mitigates mitochondrial damage and reduces serum glucose levels, ultimately facilitating the repair of the corpus cavernosum and the restoration of erectile function in a model of diabetes‐related erectile dysfunction [185].

## 7 Mitochondrial Gene Editing and Delivery by Nanomaterials

### 7.1 Strategies for Mitochondrial Genome Editing

As previously mentioned, mtDNA exhibits a higher mutation rate than nuclear DNA. mtDNA mutations progressively accumulate during normal aging and in various disease states. Pathogenic mutations in mtDNA lead to two different conditions: heteroplasmy and homoplasmy. In the heteroplasmic state, both pathogenic mtDNA with mutations and wild‐type mtDNA exist. Clinical symptoms manifest only when the heteroplasmy level exceeds a critical threshold [186]. In the homoplasmy state, only pathogenic mtDNA with mutations exists, without wild‐type mtDNA. Mitochondria can address only certain types of lesions and often degrade damaged molecules. This fundamental limitation restricts the applicability of conventional gene editing tools, which predominantly harness the intrinsic repair pathways of the nucleus. The presence of a high copy number of circular genomes per cell and critical delivery barriers also hamper the therapeutic application of gene editing tools in mitochondria.

To modify the course of diseases caused by mtDNA mutations, two primary gene‐based strategies have been developed. The first strategy involves direct intervention within the mitochondria, while the second strategy employs allotopic expression, in which specific genes are inserted into the nuclear genome. The resulting proteins, equipped with MTS, are then synthesized in the cytosol and imported into mitochondria through the endogenous protein import machinery.

For heteroplasmic mtDNA mutations, the primary therapeutic objective is the reduction of the mutant load below the pathogenic threshold. Current strategies can be broadly classified into two approaches according to their molecular targets. The first approach involves direct manipulation of mtDNA, which includes two distinct mechanisms: (1) selective degradation of mutant mtDNA by using programmable nucleases, such as zinc‐finger nucleases and transcription activator‐like effector nucleases, to eliminate mutated genomes and (2) direct correction of point mutations using precision mtDNA base editors (e.g., DddA‐derived cytosine base editors and transcription activator‐like effector‐linked deaminases). The second major strategy focuses on changes at the RNA level, employing mitochondrial RNA (mtRNA) editing to skew the transcript pool in favor of wild‐type sequences, thereby functionally compensating for the genetic defect at the transcriptional level. This RNA‐level intervention includes several technical approaches aimed at modulating the mtRNA pool, such as the use of antisense oligonucleotides (ASOs), mtRNA interference (mitoRNAi), and RNA supplementation.

For homoplasmic mtDNA mutations, therapeutic strategies must resolve the uniform genetic error present across the entire mitochondrial genome. The two most promising strategies are allotopic expression and direct mtDNA editing. Allotopic expression bypasses the defective mitochondrial gene by expressing a wild‐type, nuclear‐optimized version from the nuclear genome. A conceptually related and clinically promising strategy is nuclear‐encoded mitochondrial gene supplementation, which is currently being evaluated in clinical trials for diseases such as LHON [187]. Alternatively, mitochondrial base editors have the potential to directly correct the pathogenic mutation at its source.

### 7.2 Nanomaterials Modulating mtDNA

The targeted delivery of negatively charged oligonucleotides into mitochondria is extremely challenging due to various barriers: the double membrane, restrictive negative membrane potential, and alkaline matrix [188]. Therefore, emerging research studies employ nanomaterials to overcome these limitations.

Nanomaterials can be engineered as delivery platforms to transport ASOs, RNAi, and RNA supplementation into mitochondria. In recent years, exosomes, which are nanoscale, naturally occurring EVs, have been utilized for biotechnology applications in oligonucleotide delivery.

The D‐arm is a 2ʹ‐O‐methyl‐modified RNA sequence attached to the 5ʹ‐end of oligonucleotides to facilitate their delivery. Exosomes from fetal bovine serum can be used as a simple and biologically compatible agent to deliver D‐arm ASOs to cellular mitochondria, leading to target protein knockdown [189]. MITO‐Porter systems have been engineered for the mitochondrial delivery of ASOs. The ASOs were complexed with a linear 10‐kDa polyethyleneimine (PEI) to form NPs, which were then encapsulated within the MITO‐Porter system. The carrier was further modified with the D‐arm. This design enables the MITO‐Porter system to deliver its cargo to the IMS, followed by D‐arm‐mediated import into the mitochondrial matrix [190].

Lang et al. conjugated the mitochondrial‐targeting ligand TPP to cell‐penetrating poly(disulfide)s (CPDs) to develop a mtDNA gene silencing method, termed MitoCPDs, as a mitochondrial gene delivery platform. These MitoCPDs form nanoscale complexes with oligonucleotides and leverage the unique properties of CPDs—minimized endolysosomal trapping and glutathione (GSH)‐responsive depolymerization—to achieve efficient cytosolic and subsequent mitochondrial release of their cargo. The successful delivery of siRNA and ASOs against mtDNA‐encoded proteins effectively downregulated target gene expression and impaired mitochondrial function, thus validating the efficacy of this platform [191]. She et al. developed a vascular smooth muscle cell‐targeted nanotherapeutic to knock down Arg1, a key regulator of mitochondrial integrity. The NP, NP‐siArg1, was fabricated by encapsulating Arg1 siRNA in a PLGA‐PEI core and coating it with a platelet membrane fused with α‐SMA for homotypic targeting. This approach successfully inhibited aberrant mitochondrial cristae remodeling and mtDNA release, thereby rescuing vascular dysfunction [192].

Based on the MITO‐Porter platform, β‐MEND was developed as a multifunctional envelope‐type nanodevice for pancreatic β‐cells. Combined with a mtRNA aptamer for mitochondrial delivery, this nanocarrier system can efficiently and directly transfect mitochondrial exogenous RNA into H9c2 cells, a type of rat cardiac myoblast [174]. Moreover, the optimization of its lipid composition enhanced cellular uptake in mouse skeletal myoblast (C2C12) cells. The incorporation of the KALA peptide into this system further facilitates the efficient mitochondrial delivery of genetic cargo by providing mitochondrial targeting as a mitochondrial signal, synergistically enabling endosomal escape, and enhancing membrane fusion through mitochondrial membrane destabilization [193].

The MitoScript platform was engineered to mimic the multidomain architecture of natural mitochondrial transcription factors. It was synthesized by co‐assembling MPP and DNA‐binding hairpin pyrrole‐imidazole polyamide (PIP) oligomers into a single ultrasmall (2–3 nm) nanocluster. The PIP oligomers function as mtDNA‐binding domains for site‐specific transcription regulation. By utilizing specific mtDNA‐binding domains targeting the minor groove of TFAM light‐chain binding sites, the MitoScript platform enables selective gene suppression while providing innate near‐infrared (NIR) fluorescence for mitochondrial tracking [194].

Nanomaterials can facilitate the delivery of exogenous mtDNA into cells to replenish or replace defective genomes. Wilson et al. developed an mtDNA‐containing NP by complexing intact mtDNA, isolated from an immortalized cell line, with a cell‐penetrating peptide. The complex was conjugated with Rhodamine 123 for mitochondrial targeting and was engineered with non‐natural D‐amino acids to confer protease resistance and reduce immunogenicity. Following cellular uptake, these NPs localized to mitochondria and facilitated the retention of exogenous mtDNA for at least 4 weeks after a single administration in mitochondria‐depleted ARPE‐19 cells [195]. Chiang et al. developed mitochondria‐targeted wireless charging gold NPs (WCGs) to treat LHON. The WCGs comprise a conductive gold nanoparticle (GNP) core functionalized with PEI for transfection, TPP for mitochondrial targeting, and polyglutathione to mitigate oxidative stress and stabilize the construct. Following exposure to a high‐frequency magnetic field, the electromagnetic GNP core generates eddy currents, enabling a novel in situ magnetoelectric effect. This effect facilitates gene delivery through electroporation, enhancing the transfection of the normal *hND4* gene into retinal ganglion cells. In vitro and in vivo studies demonstrated that this strategy successfully restored mitochondrial function, prevented neuronal degeneration, and improved visual function [196].

### 7.3 Nanocarrier‐Facilitated mtDNA Transfer and Transplantation

Although mitochondrial transplantation can confer transient benefits to mitochondrial metabolism, the absence of measurable long‐term mtDNA transfer limits its therapeutic potential for application in mtDNA replacement strategies. Following several reports that mtDNA can be transferred intercellularly [129, 197, 198], an increasing number of research studies are focusing on mtDNA transfer for therapeutic applications.

Tissue‐derived, mitochondria‐rich EVs enhanced mitochondrial biogenesis and attenuated mitochondrial damage in recipient cells through mitochondrial genome transfer [199]. Oxidative stress can deplete both TFAM and mtDNA in MSCs isolated from aged or diabetic individuals. Zhao et al. observed that EVs derived from MSCs of healthy individuals attenuated mitochondrial damage in renal injury by transferring TFAM mRNA and mtDNA to recipient cells. This transfer restores TFAM protein and nucleoid stability, which revives mtDNA integrity, rescues OXPHOS function, and prevents inflammatory cytosolic mtDNA leakage, thereby highlighting the therapeutic potential of MSC‐EVs for mitochondrial‐related diseases [200].

Plant cells release nanoscale vesicles termed plant‐derived nanovesicles. These vesicles carry a diverse cargo of components, including RNA and DNA. Nanovesicles from *Artemisia annua* can deliver *A. annua*‐derived mtDNA into tumor‐associated macrophages (TAMs). This transferred mtDNA activates the cGAS‐STING signaling pathway, reprogramming the macrophages and subsequently enhancing a cytotoxic T‐cell response that promotes tumor regression [201].

### 7.4 Regulated mtDNA Release Through Nanomaterials

mtDNA‐induced inflammation is known to be associated with various inflammatory diseases. Additionally, mtDNA clearance can modulate the inflammatory manifestations of autoimmune diseases such as autoimmune encephalomyelitis and psoriasis [202]. Based on the inflammatory properties of mtDNA, some research studies constructed nanomaterials to regulate mtDNA release.

Guo et al. engineered a nanoagonist (DZ@A7) that orchestrates mtDNA release to potentiate antitumor immunity. This system is based on NIR‐triggered, mitochondria‐targeted ROS generation coupled with polydopamine‐mediated GSH depletion to induce oxidative stress and prompt mtDNA escape into the cytosol. This released mtDNA serves as a potent ligand for the cGAS‐STING pathway, an effect synergistically amplified by a co‐delivered hypoxic drug to enhance cytosolic DNA accumulation. Consequently, this targeted mtDNA release robustly activates STING signaling, leading to dendritic cell maturation and cytotoxic T‐cell infiltration for potent primary and abscopal antitumor responses [203]. Tian et al. synthesized a TPP‐ functionalized novel amphiphilic phenolic polymer for mitochondrial targeting, which contained Chlorin e6 for ROS generation and photodynamics and furan for DNA cross‐linking. The phenolic polymer induces mtDNA damage by synergizing excessive ROS with furan‐mediated mtDNA cross‐linking. The resulting excessive mtDNA damage promotes the cytosolic release of mtDNA fragments, which activate the cGAS‐STING signaling pathway in TAMs. Subsequently, the polymer was self‐assembled with Mn^2+^, a STING agonist, to form a metal‐phenolic nanomaterial, ultimately eliciting potent systemic antitumor immunity [204].

Che et al. developed a MIP nanosystem to trigger mtDNA release into the fibrotic microenvironment. It can modulate the mitochondria of HSCs under light and induce MOMP. The released mtDNA is captured by macrophages, leading to the activation of the STING signaling pathway. This activation prompts a shift in macrophage polarization toward a reparative phenotype and stimulates downstream immunomodulatory transcriptional programs. Ultimately, this cascade of events causes complete reversal of liver fibrosis, restoring the liver tissue to its normal histological architecture [172].

Some studies attempted to decrease mtDNA release to reduce inflammation and its corresponding consequences. A biomimetic NP strategy was developed to mitigate mtDNA‐mediated inflammation in spinal cord injury (SCI). Mfn2 is an essential component for axonal mitochondrial movement, functioning within the microtubule transport system where it engages directly with the Miro/Milton complex [205]. The construct MSNs‐MASM7@MI delivers MASM7 to upregulate Mfn2 in microglia, thereby restoring impaired mitochondrial dynamics. Mfn2 upregulation significantly reduces mtDNA release and prevents subsequent activation of the cGAS‐STING signaling pathway, leading to the attenuation of neuroinflammation and improvement of functional outcomes after SCI [24]. Li et al. conceptualized and developed a nanoscale mtDNA scavenger (Nanosweeper [NS]) to downregulate mtDNA released after cardiac injury to decrease inflammatory responses that lead to heart failure. The NS includes the peptide APWHLSSQYSRT (CTP, a myocardial cell targeting unit) to target myocardial cells, the peptide FFVDF to extend the retention time and stability of the NS through transformation, SYBR Green I as an mtDNA fluorescent probe to target mtDNA and facilitate real‐time imaging, and C‐176 as a STING inhibitor. This system leverages the unique properties of nanomaterials to promote their accumulation in lysosomes following cellular uptake. The NS can target and bind mtDNA while simultaneously releasing C‐176, ultimately transporting the NS‐mtDNA complex to lysosomes for degradation. The NS effectively attenuated myocarditis‐ associated inflammatory cascade [206].

## 8 Approaches to Mitochondrial Transfer and Transplantation

### 8.1 Attempts for Mitochondrial Transfer and Transplantation

For diseases induced by damaged mitochondria, replenishment with healthy mitochondria has been proposed as a promising therapeutic strategy. As a result, the utilization of mitochondrial transfer and transplantation to achieve this goal has received increasing interest from researchers.

Intracerebellar transplantation of healthy mitochondria significantly attenuated progressive Purkinje cell loss and ameliorated behavioral impairment in a mice model of cerebellar neurodegeneration. The therapeutic benefits were attributed to improved mitochondrial function and a reduction in Parkin‐dependent mitophagy and apoptosis in Purkinje cells [158]. The administration of exogenous mitochondria to doxorubicin‐damaged neonatal rat cardiomyocytes substantially enhanced the function of these cardiomyocytes. Following mitochondria transplantation, ROS production and lipid peroxidation decreased, concomitant with the restoration of MMP and increased production of ATP and GSH. Consequently, apoptotic and necrotic cell death rates were significantly suppressed. Thus, this therapy could be a promising strategy to mitigate cancer treatment‐related complications [207].

In MASH models, the transplantation of healthy mitochondria increased the intracellular levels of NAD^+^, a crucial sirtuin 1 cofactor, thereby activating pathways that mitigate oxidative stress, autophagy, and inflammation in vivo and in vitro [208]. Yu et al. successfully delivered BMSC‐derived mitochondria to chondrocytes in vitro and in vivo, leading to improved osteoarthritis (OA) phenotypes, enhanced mitochondrial function, and inhibition of apoptosis. The authors also found that these mitochondria activated PGC‐1α‐mediated biogenesis, demonstrating a dual‐action mechanism that positions BMSCs as an effective mitochondrial donor for ameliorating knee OA [147].

The administration of M2‐like macrophages could protect against doxorubicin‐induced cardiotoxicity by transferring mitochondria to damaged cardiomyocytes [209]. In the study of Zhang et al., bone marrow‐derived macrophages were modified to mitochondria‐augmented macrophages (MTMs), which exhibit the characteristics of M2‐like macrophages, following co‐culture with mitochondria. The co‐culture method enhanced the migratory, invasive, and phagocytic capacities of these macrophages. These mitochondria‐treated macrophages were intravenously injected into a mouse model of myocardial infarction. A portion of the transplanted mitochondria was released from MTMs, which were subsequently internalized by cardiomyocytes; thus, this therapy improved cardiac function and attenuated cardiac remodeling by reducing fibrosis and promoting angiogenesis [210]. In another study, researchers transplanted bone marrow from wild‐type mice to *Ndufs4* ^−/−^ mice. They found that transplanted bone marrow‐derived mitochondria were transferred to diseased host cells, leading to alleviation of LS. The administration of mitochondria isolated from mouse livers and human mitochondrial transplantation also increased the survival of *Ndufs4* ^−/−^ mice [211]. Mitochondrial transplantation into adipose‐derived stem cells (ADSCs) generated “Mito‐ADSCs” with enhanced metabolic function and a pro‐angiogenic secretory profile. Following their delivery through a decellularized adipose tissue hydrogel, these modified cells synergistically promoted robust angiogenesis and adipose tissue regeneration [212].

### 8.2 Nanomaterial‐Facilitated Mitochondrial Transfer and Transplantation

Nanomaterials integrated with multiple components can perform complex roles in the application of mitochondrial transplantation and transfer. Nanomaterials can enhance the targeting ability for exogenous mitochondria. A common strategy is using nanomaterials to endow cells or organelles with targeting capability for mitochondrial transplantation or transfer [144, 213]. Nanomaterials can also enable multi‐step cascade targeting. Wu et al. developed an engineered mitochondrial nanomotor with stepwise targeted delivery ability. The nanomotor was constructed by coating mitochondria with N‐methacryl‐L‐arginine (M‐Arg) through polymerization, forming M/PMA, which was further modified with denatured bovine serum albumin (BSA). This BSA layer enables the construct to hitchhike on activated neutrophils in circulation by specific binding to FcγR expressed on their surface. After reaching the inflammatory site of myocardial I/R injury, the neutrophils undergo formation of neutrophil extracellular traps (NETosis)—triggered by DAMPs from necrotic cardiomyocytes—releasing the carried M/PMA/BSA through these traps. In the extracellular environment, the nanomotors actively migrate toward inducible nitric oxide synthase (iNOS) expressed by damaged cardiomyocytes, achieving precise mitochondrial delivery and repair. The authors used an immediate local injection during reperfusion to ensure an initial high mitochondrial concentration, followed by intravenous administrations to support sustained myocardial repair; this approach showed fewer side effects compared to application through intramyocardial injection alone. This combination of neutrophil‐mediated hitchhiking and controlled, stage‐matched delivery is tailored to the pathophysiology of myocardial I/R injury and has potential to advance mitochondrial transplantation toward clinical translation [149].

In Figure 5, we highlight various nanomaterial‐based strategies to enhance the efficiency of mitochondrial transfer and transplantation. Apart from mitochondrial applications, another strategy to improve mitochondrial function is constructing nanomaterial‐based artificial ATP synthesis systems that can generate ATP. The details of related research studies are presented in Table 3.

**FIGURE 5:**
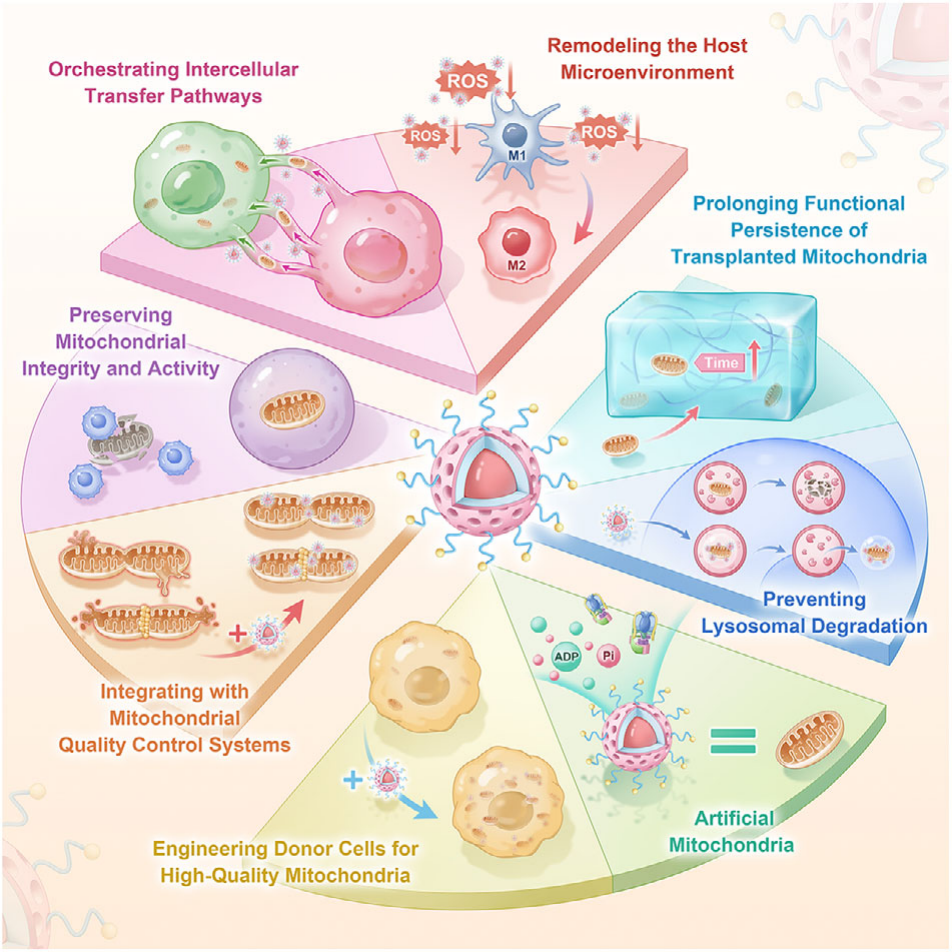
Nanomaterial‐enabled strategies for mitochondrial transplantation and transfer. Nanomaterials offer innovative and precise solutions to overcome the key challenges in mitochondrial transplantation and intercellular transfer, as shown by eight complementary strategies: (1) orchestrating intercellular transfer pathways: functionalized nanoparticles (NPs) can stimulate or mimic natural transfer conduits to directionally enhance mitochondrial delivery efficiency between cells; (2) preserving mitochondrial integrity and activity: nanocarriers provide a protective physicochemical microenvironment during mitochondrial isolation and transit; (3) remodeling the host microenvironment for improved engraftment: nanomaterials modulate local immune responses, reduce oxidative stress, and provide supportive bioenergetic cues to enhance transfer and transplant efficiency; (4) integrating with mitochondrial quality control systems: NPs are engineered to restore impaired mitophagy and enhance mitochondrial biogenesis; (5) engineering donor cells for high‐quality mitochondria: nanomaterials engineer donor cells to upregulate mitochondrial production, function, or stress resistance prior to extraction and transfer; (6) prolonging functional persistence of transplanted mitochondria: sustained‐release nanoformulations extend the bioenergetic lifespan of transplanted organelles; (7) preventing lysosomal degradation and promoting cytosolic release: nanomaterials facilitate mitochondria released directly into the host cytoplasm to evade lysosomal destruction; and (8) biomimetic nanomaterials as artificial mitochondria: nanomaterials mimic core mitochondrial functions, providing a cell‐free alternative therapy.

**TABLE 3:** Nanomaterial application in mitochondria transplantation and transfer.

Reference	Materials	Disease condition	Strategies	Details	Results
[149]	M/PMA/BSA	Myocardial I/R injury	Targeted delivery	Combining neutrophil hitchhiking with nanomotor chemotaxis as an active delivery system to transplant mitochondria derived from huMSCs to cardiomyocytes	Successfully targets injured cardiomyocytes, supplements energy, and enhances cardiac function
[144]	Mitochondria coated with Dextran‐TPP	Chemobrain and neuropathy	Targeted delivery	Nasal administration of mitochondria coated with dextran‐TPP polymer to deliver stem cell‐derived mitochondria to meningeal macrophages	Reverses cisplatin‐induced cognitive deficits and resolves neuropathic pain at a lower dose compared to uncoated mitochondria
[213]	RGD‐mitoEVMs	Post stroke	Targeted delivery	Mitochondria derived from MSCs are delivered via EVs, targeting injured endothelial cells via RGD recognition of integrins αvβ3	MSC‐derived mitochondria promote tip cell transition, stimulate angiogenesis after stroke, alleviate brain atrophy, and improve functional rehabilitation
[177]	NBP@PLGA‐HA, with intraperitoneal injection of L‐778123	Cancer	Orchestrating Intercellular Transfer Pathways	NBP@PLGA‐HA blocks energy supply to tumor cells, and intraperitoneal injection of L‐778123 inhibits mitochondrial transfer from CTLs to 4T1 cells via nanotubes	Inhibits primary tumor growth and significantly suppresses distant metastasis
[214]	MTBG	Aged bone regeneration	Orchestrating Intercellular Transfer Pathways	Released melatonin potently stimulates TNTs formation between BMSCs, facilitating mitochondrial transfer from healthy to senescent BMSCs	Effectively reverses cellular senescence by restoring mitochondrial function, stabilizing membrane potential, and boosting ATP synthesis
[215]	FluidFM‐based approach	_	Orchestrating Intercellular Transfer Pathways	Integrate atomic force microscopy, optical microscopy, and nanofluidics to enable force‐controlled, real‐time, minimally invasive manipulation for the precise extraction and transplantation of organelles	Transplanted mitochondria not only fused with the host network but also propagate their mtDNA over subsequent cell divisions
[26]	IONP‐engineered hMSCs	Pulmonary fibrosis	Orchestrating Intercellular Transfer Pathways	hMSCs combined with IONPs upregulate Cx43 expression to facilitate mitochondrial transfer from hMSCs to injured lung cells	Achieves remarkable mitigation of fibrotic progression due to promoted intercellular mitochondrial transfer in a mouse model of pulmonary fibrosis
[27]	Pg‐Fe‐hMSC	Pulmonary fibrosis	Orchestrating Intercellular Transfer Pathways and Engineering Donor Cells for High‐Quality Mitochondria	After activating mitochondrial biogenesis in human MSCs with pioglitazone, IONPs are added to upregulate Cx43 expression, increasing mitochondrial transfer from MSCs to injured lung cells	Restores mitochondrial homeostasis, reactivates inhibited mitophagy, and consequently recovers impaired cellular functions
[166]	PGA‐Cu‐S@G	Discogenic pain	Orchestrating Intercellular Transfer Pathways, Remodeling the Host Microenvironment, and Preventing Lysosomal Degradation	A multifunctional composite employs SS‐05 for targeting, GAP134 for Cx43 upregulation, and PGA‐Cu for ROS scavenging. This system promotes M2 macrophage polarization and utilizes pH‐buffering for lysosomal escape, thereby facilitating mitochondrial transfer from macrophages to both nucleus pulposus cells and neurons	Effectively preserves the intervertebral disc height, maintains the structural integrity of nucleus pulposus cells, and restores pain thresholds
[216]	Photothermal nanoblade	_	Orchestrating Intercellular Transfer Pathways	Mitochondria are transplanted using a nanoscale titanium film and a uniquely designed tip	Effectively restores respiratory function and metabolic homeostasis in recipient cells
[217]	Mitochondria‐rich EVs	Ischemic myocardium	Preserving Mitochondrial Integrity and Activity	Mitochondria‐rich EVs are isolated via ultracentrifugation from the conditioned medium of human induced pluripotent stem cell‐derived cardiomyocytes	EV encapsulation confers enhanced resilience against combined calcium overload and oxidative stress
[218]	AM‐mito	Focal cerebral I/R injury	Preserving Mitochondrial Integrity and Activity	Coating mitochondria with cationic DOTAP mixed with DOPE endows a positive surface charge to improve internalization and protect transplanted mitochondria derived from huMSCs	Improve mitochondrial internalization and neuroprotection in neurons in vitro. Intravenous infusion of AM‐mito immediately after focal cerebral I/R amplified cerebroprotection in vivo
[25]	Mito@euMVs	Chronic wound	Preserving Mitochondrial Integrity and Activity and Prolonging Functional Persistence of Transplanted Mitochondria	Enucleated MSCs‐derived microvesicles containing functional mitochondria are delivered transdermally into fibroblasts and HUVECs using soluble PVA microneedle patches	Inhibits and rejuvenates hyperglycemia‐induced cellular senescence
[219]	(PEG‐DET/HSP27)/EV	Ischemic stroke	Preserving Mitochondrial Integrity and Activity	EVs protect mitochondria, and HSP27 preserves the tight junction integrity of brain endothelial cells	Increasing brain endothelial cells’ metabolic function and tight junction integrity
[220]	MIT@ZIF‐8	Cancer	Preserving Mitochondrial Integrity and Activity and Preventing Lysosomal Degradation	Isolated mitochondria are encapsulated within ZIF‐8 and conjugated with PEI to achieve lysosomal escape, with Tat for cell penetrating, to transplant mitochondria from non‐tumorigenic cells into cancer cells	Result in notable tumor‐suppressive effects
[221]	DN‐Mito	RA	Preserving Mitochondrial Integrity and Activity, Remodeling the Host Microenvironment and Prolonging Functional Persistence of Transplanted Mitochondria	Prussian blue nanozymes neutralize excess ROS in the RA microenvironment, while a hydrogel protects mitochondria and provides sustained support, facilitating BMSCs‐derived mitochondria transplantation to macrophages	Efficiently scavenges ROS to alleviate inflammation, leverages mitochondrial transfer to restore cellular energy metabolism, promotes chondrocyte proliferation and differentiation, alleviates RA symptoms, and repairs joint tissue in vivo and in vitro
[165]	Mito‐PB	IVDD	Targeted delivery and Remodeling the Host Microenvironment	Mitochondria isolated from rat cardiomyocytes are decorated with mitochondrial‐targeting SS‐31 and antioxidative/anti‐inflammatory PBE to transplant mitochondria isolated from rat cardiomyocytes to nucleus pulposus cells through intradiscal injection	Improves mitochondrial quality control in NPCs, significantly delays IVDD progression in rats, and demonstrates efficacy in alleviating pathological abnormalities
[161]	Pep‐Mito	Myocarditis and Cardiac Fibrosis	Targeted delivery and Remodeling the Host Microenvironment	Functionalized mitochondria derived from healthy hearts are decorated with bifunctional peptide SDKP and SS‐31 for uptake by neutrophils	Significantly attenuates inflammation and oxidative stress through i.v. mitochondrial transplantation, ameliorates myocardial injury, improves cardiac function, and alleviates fibrosis and heart failure during the chronic cardiomyopathy phase in mice with EAM
[29]	a127/mito@ZIF@Ma	Acute Lung Injury	Remodeling the Host Microenvironment and Preventing Lysosomal Degradation	Macrophage membranes encapsulate anti‐inflammatory miRNA‐127 antagonists and healthy mitochondria, with pH‐responsive ZIF‐8 facilitating lysosomal escape	Promotes macrophage polarization from M1 to M2 phenotype and alleviates lung inflammation in ALI
[222]	CM/NM/Mito@Cap	Ischemic heart disease	Remodeling the Host Microenvironment	huMSCs‐derived mitochondria are delivered using NO‐releasing nanomotors and cardiomyocyte membrane fragments to target injured heart tissue. A ROS‐responsive diselenide cross‐linker scavenges ROS, and released NO modulates the inflammatory and oxidative microenvironment for delivery to H9c2 cells	Restores cardiac function and maintains it for 2 weeks after discontinuation
[223]	HQ‐Mitofactories	Spinal cord injury	Remodeling the Host Microenvironment	Hollow mesoporous Prussian blue coated with PDA is anchored on the surface of MSCs to scavenge ROS and facilitate mitochondria transfer from MSCs to neurons	Successfully recovers locomotor function within 4 weeks in mouse models, with 40% fully restoring walking after hindlimb paralysis
[224]	nMITO	Acute myocardial injury, liver injury, and pancreatitis	Targeted delivery and Remodeling the Host Microenvironment	Combines the injury‐targeting and immunomodulatory properties of neutrophil membranes to transplant mitochondria derived from cardiac cells to HUVECs and injured tissue cells	Effectively attenuates inflammation and promotes tissue repair
[225]	WOC	Diabetic wound	Remodeling the Host Microenvironment	Leverages the anti‐inflammatory properties of COS to polarize macrophages toward the M2 phenotype, facilitating mitochondrial transfer from M2 macrophages to endothelial cells	Restores vascular function through upregulated angiogenesis genes, enhanced migration, and tube formation, accelerates wound closure, resolves inflammation, and promotes scarless regeneration in diabetic rat models
[226]	Silicon‐based treatment	Diabetic bone defects	Integrating with Mitochondrial Quality Control Systems	Silicon enhances mitochondrial function in macrophages and promotes the packaging of functional mitochondria into microvesicles, thereby facilitating mitochondrial transfer from macrophages to endothelial and neuronal cells	Significantly promotes vessel formation, nerve growth, and mineralized tissue development
[227]	mNP‐Mito	LHON	Integrating with Mitochondrial Quality Control Systems	Adhesion of PARKIN mRNA‐loaded Lipofectamine 2000 nanoparticles to healthy mitochondria maintains exogenous mitochondrial activity and promotes mitophagy of damaged mitochondria, facilitating mitochondrial transplantation in vivo and in vitro	Enhances the presence of healthy mitochondria, sharply increases complex I activity (76.5% vs. 29.5% in Rot damaged group), and greatly mitigates ocular mitochondrial disease‐related phenotypes
[171]	Mito‐MEN	Pulmonary fibrosis	Integrating with Mitochondrial Quality Control Systems	Healthy mitochondria from heart tissue are anchored to nanoparticles loaded with Parkin mRNA, then transplanted to A549 cells in vitro and AECs in vivo	Ultimately restores mitochondrial function and alleviates pulmonary fibrosis phenotypes
[228]	FMC‐MT	Bone defect	Engineering Donor Cells for High‐Quality Mitochondria and Preventing Lysosomal Degradation	Selects MG63 cells as a mitochondrial source for transplantation, combining with FMC for delivery to MSCs	Demonstrates superior ability to direct MSCs toward an osteogenic fate compared to MSC‐derived mitochondria and improves bone regeneration
[30]	Met‐EVs@DAM/HAMA‐MNP	Chronic wound	Engineering Donor Cells for High‐Quality Mitochondria and Prolonging Functional Persistence of Transplanted Mitochondria	Metformin enhances mitochondrial biogenesis and increases secretion of mitochondria‐containing EVs. A hydrogel microneedle patch gradually releases these EVs into wounded tissues	Contributes to the restoration of mitochondrial membrane potential, elevation of ATP levels, and reduction in ROS production, collectively improving cellular bioenergetics and mitigating oxidative stress in the injured tissue
[229]	MitoFactory	Mitochondrial damage induced via various treatments: Doxorubicin, Antimycin A, CCCP	Engineering Donor Cells for High‐Quality Mitochondria	MoS_2_ nanoflowers with atomic‐scale vacancies stimulate the expression of genes associated with mitochondrial biogenesis to facilitate transfer from MSCs to injured cells	Improves mitochondrial respiratory capacity and ATP production in recipient cells, achieving remarkable restoration of cell function in models of mitochondrial damage
[28]	CAP	LHON	Engineering Donor Cells for High‐Quality Mitochondria	Uses CAP to form stable complexes with plasmid DNA encoding CD38 to generate “super donor” MSCs, then facilitates mitochondrial transfer from MSCs to GM10742 cells	Rescues mtDNA defects and alleviates LHON‐associated symptoms in LHON male mice
[230]	MSC‐loaded GelMA	Surgical brain injury	Prolonging Functional Persistence of Transplanted Mitochondria	A GelMA hydrogel functions as a local reservoir, drastically reducing rapid clearance and enabling prolonged MSC retention to facilitate mitochondrial transfer from MSCs to HT22 cells	Attenuates brain edema, reduces inflammation, and enhances neurological function
[231]	FMC‐MT	Osteoarthritis	Preventing Lysosomal Degradation	FMCs encapsulating mitochondria isolated from hMSCs deliver them to chondrocytes via membrane fusion, bypassing the endocytic pathway	Enable faster and more efficient delivery to chondrocytes compared with naked mitochondria, reduces inflammatory cytokines and MMP13, increases extracellular matrix components, and promotes cartilage regeneration in vitro and in vivo
[34]	GC@SiO2	_	Artificial mitochondria	A mesoporous silica nanocapsule co‐loaded with GOx and CAT creates a transmembrane proton gradient, which drives ATP synthesis via an embedded ATP synthase within a lipid bilayer	Supports NADH biosynthesis and glucose‐powered OXPHOS, mimicking key metabolic features of living mitochondria
[33]	UCTR	AKI	Artificial mitochondria	Cloaked with membranes from activated renal tubular epithelial cells for targeted delivery, UCNPs convert NIR‐II light to activate photosynthesis within the artificial mitochondria, generating ATP and NADPH	Ameliorates hypoxia‐induced energy deficits, activates the AMPK/PGC‐1α pathway to restore membrane potential, and synergistically accelerates tubular repair
[31]	SiO2@Au‐ACP	_	Artificial mitochondria	Integrates ACP and ATP synthase with plasmonic Au clusters for ATP generation and ROS scavenging	Effectively enhances the stress resistance of ATP synthase‐based energy generating systems
[32]	AMNs	Ischemic heart disease	Artificial mitochondria	Comprises a motion unit (M‐Arg) for chemotactic targeting, an energy‐generating unit (PCr) to regenerate ATP, and a barrier‐crossing unit (PFA) for mucus penetration and intestinal absorption	200 µg AMNs emulate 5 × 10^6^ natural mitochondria, restoring energy metabolism and structural function in damaged hearts at the genetic level

#### 8.2.1 Orchestrating Intercellular Transfer Pathways

As mitochondrial transfer occurs through various pathways, improving the quality and quantity of these pathways can impact transfer efficiency. For instance, overexpressing Miro1 [102, 232]; downregulation of the neuronal guidance protein splice variant MICAL2PV [233]; ROCK inhibition [103, 234]; and introduction of exogenous thymosin beta‐4 (Tβ4) [235], Epimedin C [236], and magnolol [237] can significantly promote the efficiency of mitochondrial transfer through adjustment of TNT formation.

The indole hormone melatonin (N‐acetyl‐5‐methoxytryptamine [Mel]) is secreted by the pineal gland. Mel promotes TNT formation and mitochondrial transfer among neuronal cells. Mel is also involved in antioxidant activity, mitochondrial biogenesis, and protection against oxidative stress‐induced damage [238, 239]. Mel‐loaded microspheres (MTBGs) were fabricated by leveraging the mesoporous structure of bioactive glass (BG) for drug delivery. The released Mel potently stimulated the formation of TNTs, which subsequently facilitated intercellular mitochondrial transfer between healthy and senescent BMSCs. This process effectively reversed cellular senescence by restoring mitochondrial function, stabilizing MMP, and enhancing ATP synthesis [214].

CX43 disruption inhibits mitochondrial transport from BMSCs to pulmonary alveoli [240]. By upregulating CX43 and promoting the formation of CX43‐containing GJ channels (GJCs), iron oxide NPs (IONPs) enhance mitochondrial transfer between human MSCs and injured AECs [26, 27]. GAP134, a small‐molecule GJC modifier, can upregulate CX43 expression and promote GJC assembly [241]. Because CX43 enables the propagation of long‐distance signals across cells by establishing functional GJCs at the end of TNTs, CX43 regulation subsequently influences the formation and function of TNTs by modulating the extended membrane terminals that constitute these intercellular connections [242]. Following functionalization with GAP134, NPs exhibit enhanced intercellular mitochondrial transfer efficiency in macrophage donor cells and injured nucleus pulposus cells (NPCs) and neurons through TNTs, thus indicating a promising therapeutic approach for managing intervertebral disc degeneration (IVDD) [166].

Another strategy is the use of composite nanomaterials functionalized with mitochondrial transfer regulators. NBP@PLGA‐HA, synthesized using PLGA, nebivolol hydrochloride, 3‐bromopyruvate, and CD44, could bidirectionally block the energy supply of tumor cells [177]. An intraperitoneal injection of L‐778123, a dual inhibitor of farnesyltransferase and geranylgeranyltransferase 1, inhibited mitochondrial transfer from cytotoxic T lymphocytes (CTLs) to tumor cells through nanotubes, thus blocking the additional respiratory pathway of tumor cells and depleting CTLs.

In addition to facilitating the current natural mechanisms of mitochondrial transfer, some research studies constructed artificial pathways for mitochondria transfer. The photothermal nanoblade introduced a paradigm for organelle transplantation by using a nanoscale titanium film and a uniquely designed tip (3 µm inner diameter). This wide orifice was crucial for delivering sizable mitochondria without clogging or shear‐induced damage. A localized laser pulse vaporizes the surrounding medium, inducing a cavitation bubble that temporarily disrupts the plasma membrane, thereby enabling direct cytosolic delivery of mitochondria under pressure. Following their isolation from donor cells, mitochondria are loaded and transferred by the photothermal nanoblade directly into recipient cells. This approach effectively restored respiratory function and metabolic homeostasis in recipient cells [216].

Under in vitro conditions, the FluidFM‐based approach enables precise extraction and transplantation of organelles, including mitochondria, in living cells with subcellular resolution. This technology integrates atomic force microscopy, optical microscopy, and nanofluidics to achieve force‐controlled, real‐time minimally invasive operations. During extraction, applied fluidic forces reshape mitochondria to form a distinct “pearls‐on‐a‐string” morphology. For transplantation, the loaded probe is accurately positioned and inserted into recipient cells in the contact mode, allowing the targeted delivery of isolated organelles. A notable observation is that the transplanted mitochondria not only fused with the host network but also propagated their mtDNA over subsequent cell divisions, confirming stable, long‐term engraftment. This platform has immense potential for application in fundamental research and therapy in several fields, from mitochondrial biology to synthetic biology [215].

#### 8.2.2 Preserving Mitochondrial Integrity and Activity

Some studies have developed nano‐scale EVs to preserve mitochondrial integrity and activity. As reported by Ikeda et al., EV‐encapsulated mitochondria, but not free mitochondria, retained their structural and functional integrity for up to 24 h under co‐treatment with 2 mM Ca^2+^ and 100 mM H_2_O_2_, thus demonstrating that EV encapsulation confers enhanced resilience against combined Ca^2+^ overload and oxidative stress (Figure 6a) [217]. By using EVs conjugated with heat shock protein 27 (HSP27), Dave et al. constructed (PEG‐DET/HSP27)/EV to increase brain endothelial cells (BECs) through encapsulated mitochondria and preserve their tight junction integrity through HSP27 in ischemic stroke (Figure 6b) [219].

**FIGURE 6:**
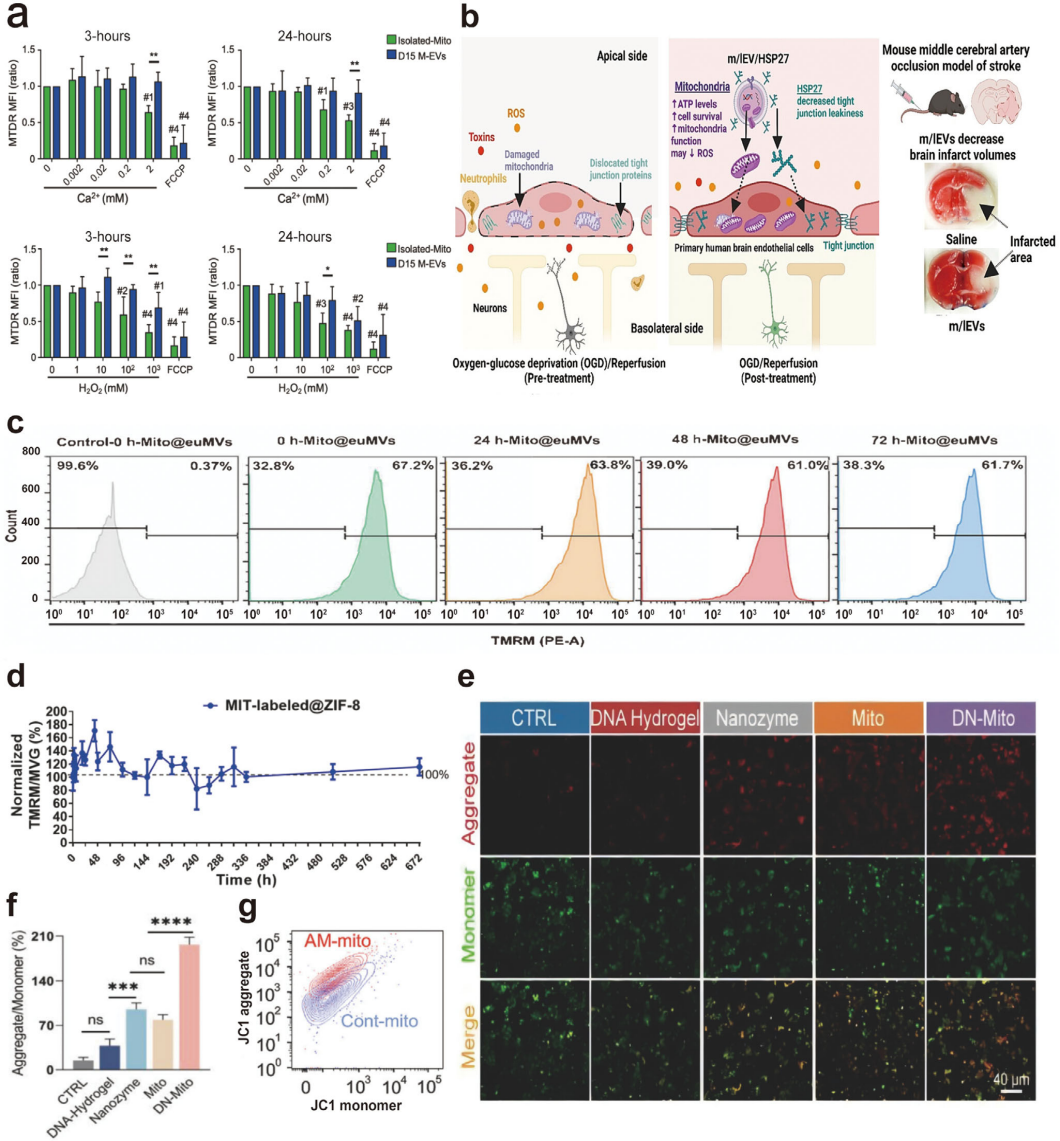
Use of nanomaterials to preserve mitochondrial integrity and activity. (a) Mitochondrial functions were evaluated by MitoTracker Deep Red for extracellular vesicles (EV)‐encapsulated mitochondria and isolated mitochondria incubated in different concentrations of Ca^2+^ and H_2_O_2_. Reproduced with permission [217]. Copyright 2021, Elsevier. (b) EV/HSP27 mixture protects brain endothelial cells in ischemic stroke. Reproduced with permission [219]. Copyright 2024, Elsevier. (c) Mitochondrial activity in Mito@euMVs assessed by flow cytometry. Reproduced with permission [25]. Copyright 2025, Wiley‐VCH GbmH. (d) The relative membrane potential of ZIF‐8‐coated mitochondria maintained for up to 4 weeks. Reproduced with permission [220]. Copyright 2025, Wiley‐VCH GbmH. (e) JC‐1 assay for assessing mitochondrial membrane potential (MMP) in LPS‐induced macrophages co‐cultured with mitochondria. (f) Quantification of JC‐1 staining results. DN‐Mito (DNA hydrogel + nanozyme + mitochondria). Reproduced with permission [221]. Copyright 2025, Elsevier. (g) Fluorescence‐activated cell sorting analysis showed that JC1 MMP became slightly higher in artificial membrane (AM)‐mito compared to that in control mitochondria. Reproduced with permission [218]. Copyright 2022, Springer Nature.

Dong et al. engineered enucleated MSC‐derived MVs containing functional mitochondria (Mito@euMVs), which maintain the activity of mitochondria up to 72 h compared to rapid inactivation of naked mitochondria (Figure 6c) [25]. The optimized vesicle size of 3.0 µm diameter was larger than the size of mitochondria, which maximized encapsulation efficiency. Eliminating nuclei from MSCs prior to vesicle generation reduced the nuclear content in the resultant MVs, thereby significantly enhancing the safety of Mito@euMVs. Mito@euMVs effectively delivered functional mitochondria into both fibroblasts and human umbilical vein endothelial cells (HUVECs), thereby attenuating and even reversing cellular senescence induced by hyperglycemia in chronic wounds [25].

However, EVs exhibit a short circulation half‐life in vivo, with clearance typically occurring within minutes to a few hours [243, 244]. The clearance is predominantly mediated by the mononuclear phagocyte system, particularly in organs such as the liver and spleen [245]. Incorporating factors that reduce phagocytosis by macrophages, such as CD47, has been shown to prolong the circulation time of EVs [245, 246]. The use of engineered EVs represents another promising strategy.

Scaffold platforms provide protection for mitochondria and EVs. Encapsulation within metal‐organic frameworks (MOFs) can protect mitochondria and maintain their bioactivity for 4 weeks (Figure 6d) [220]. Mitochondria can also be protected by encapsulating them in a biomaterial hydrogel. Such hydrogel‐encapsulated mitochondria have demonstrated enhanced repair efficacy in damaged cells (Figure 6e,f) [221]. The use of a cationic lipid artificial membrane to coat mitochondria can protect their structure and functional integrity and improve mitochondrial internalization (Figure 6g) [218].

#### 8.2.3 Remodeling the Host Microenvironment for Improved Engraftment

In a ROS‐rich microenvironment, isolated mitochondria rapidly lose their activity. Therefore, microenvironment regulation is critical to improve the efficiency of mitochondrial transfer and transplantation. Immune conditions can be modified by exogenous mitochondria and ROS suppression. In a hindlimb ischemia mouse model, transplanted mitochondria were found to induce M2‐like polarization of macrophages [247]. By utilizing the anti‐inflammatory properties of chitosan oligosaccharide, a glucose/pH‐responsive nanoplatform (WOC) can promote diabetic wound healing by enhancing mitochondrial transfer. The WOC platform polarized macrophages toward the anti‐inflammatory M2 phenotype. These M2 macrophages subsequently served as mitochondrial donors, transferring their functional mitochondria to injured ECs through vesicles, thereby promoting angiogenesis and tissue regeneration [225]. In an alkaline aqueous solution, metal‐phenolic network NPs (PGA‐Cu) were synthesized through the self‐assembly of gallic acid (GA), leveraging its multiple adjacent phenolic hydroxyl groups. Coordination‐driven polymerization between GA and copper ions remarkably enhanced the antioxidant capacity of the resultant NPs, which demonstrated excellent ROS scavenging performance in vitro. By effectively removing excessive ROS from macrophages, these NPs help restore mitochondrial function and promote a shift in macrophage polarization toward the reparative M2 phenotype. Ultimately, this treatment promoted the transfer of mitochondria from M2 macrophages to oxidatively damaged nucleus pulposus cells and neurons. This modulation establishes a promising method to treat IVDD [166].

The nanomaterial a127/mito@ZIF@Ma carries the anti‐inflammatory miRNA‐127 antagonist as a cargo with healthy mitochondria and is camouflaged by macrophage‐derived cell membrane. Following targeting and internalization by macrophages, this nanomaterial remodeled the inflammatory microenvironment, exerting synergistic effects to rejuvenate the innate immune response in ALI [29].

Enzymes with antioxidant activity, such as Prussian blue nanozymes, are also commonly utilized. By scavenging ROS, Prussian blue nanozymes protect MSCs from producing mitochondria and promote the transfer of functional mitochondria to damaged neurons, leading to significant neuroregeneration and remyelination, thereby alleviating paralysis in SCI [223]. By using Prussian blue nanozymes, Wang et al. synthesized a polymer‐modified DNA hydrogel with functional mitochondria. This composite system neutralizes excess ROS within the RA microenvironment, thereby mitigating inflammation. Concurrently, the hydrogel facilitates efficient intracellular delivery of viable mitochondria, which inhibits ROS production at its source and promotes tissue repair, extending the therapeutic paradigm of mitochondrial transplantation into a localized, biomaterial‐guided platform [221].

The macromolecular therapy PSP was designed by integrating mitochondrial‐targeting SS‐31 and bioactive phenylboronic acid pinacol ester (PBE) motifs on a polymeric platform. This design utilizes the known antioxidative/anti‐inflammatory properties of PBE. Following intradiscal injection of PSP‐decorated exogenous mitochondria, some mitochondria were internalized into NPCs, orchestrating the transfer of therapeutic mitochondria between NPCs. This capacity acts as an advanced bio‐intervention, amplifying its effects through a transcellular pathway, which accounts for its superior performance in treating IVDD [165].

Hu et al. functionalized exogenous mitochondria using a newly synthesized bifunctional peptide SDKP‐SS31 to enhance their anti‐inflammatory and anti‐fibrotic effects in mitochondrial therapy. As an endogenous tetrapeptide, SDKP inhibits fibrosis and suppresses inflammatory cell activation by regulating TNF‐α/NF‐κB signaling, and its therapeutic efficacy has been validated across multiple cardiovascular diseases, including heart failure, myocardial infarction, and autoimmune myocarditis. Following their administration, these SDKP‐SS31‐functionalized mitochondria were efficiently taken up by neutrophils and trafficked to cardiac inflammatory sites through neutrophil recruitment. After cellular uptake, these mitochondria promoted mitochondrial fusion within the neutrophils, rescuing impaired mitochondrial function. This restoration suppressed mtPTP opening and decreased mtROS overproduction. Collectively, these actions inhibited NETosis, which subsequently attenuated the amplification of inflammation and reduced NET‐induced cardiomyocyte apoptosis. This nanomaterial system exerted potent synergistic effects in experimental autoimmune myocarditis [161].

By functionalizing mitochondria with NO‐releasing nanomotors and cardiomyocyte membrane fragments, a platform was created that chemotactically targets injured heart tissues. These nanomotors can scavenge ROS through an ROS‐responsive diselenide cross‐linker and release NO, thereby modulating the inflammatory and oxidative disease microenvironment. This targeted immunomodulation promoted the functional integration of the transplanted mitochondria, enhanced their uptake and retention by cardiomyocytes, and ultimately restored bioenergetic metabolism [222].

Zhang et al. constructed engineered neutrophil membrane‐fused mitochondria (nMITO) that combines the injury‐targeting and immunomodulatory properties of neutrophil membranes with the intrinsic cell repair functions of mitochondria. By inheriting chemokine and cytokine receptors from the neutrophil membrane, nMITO can absorb and neutralize inflammatory mediators, thereby blocking inflammatory cascades. Simultaneously, the integrated mitochondria restore cellular bioenergetics and mitigate mitochondrial dysfunction in injured cells. This dual mechanism is further enhanced by targeted delivery: nMITO selectively homes to damaged ECs through β‐integrins and can be transported through TNTs to injury sites. In murine models of acute myocardial, hepatic, and pancreatic injuries, nMITO effectively attenuated inflammation and promoted tissue repair, demonstrating its potential as a multitarget therapy for acute organ injury [224].

#### 8.2.4 Integration With MQC Systems

Mitophagy is inhibited during persistent mitochondrial damage in PF. This inhibition is attributed to the decreased expression of the Parkin protein, which impedes the effective removal of dysfunctional mitochondria. To restore impaired mitophagy that hampers mitochondrial transfer in PF, Wang et al. developed a mitophagy‐enhanced NP (Mito‐MEN). This system synchronously regulates both functional and dysfunctional mitochondrial pools by anchoring healthy mitochondria to NPs loaded with Parkin mRNA (Figure 7a). This dual approach not only delivered functional mitochondria but also promoted Parkin‐mediated degradation of damaged ones, ultimately restoring mitochondrial function and alleviating the PF phenotype in a murine model [171]. Similarly, Parkin mRNA‐loaded NP‐engineered mitochondria (mNP‐Mito) were developed to replace dysfunctional mitochondria and restore complex I activity for treating LHON. In addition to delivering functional mitochondria, it simultaneously enhanced the clearance of impaired mitochondria. In a rotenone (Rot)‐induced mouse model of complex I deficiency, mNP‐Mito treatment significantly increased complex I activity from 29.5% (Rot‐damaged group) to 76.5%, supporting the recovery of ATP production and alleviating ocular disease phenotypes (Figure 7b) [227].

**FIGURE 7:**
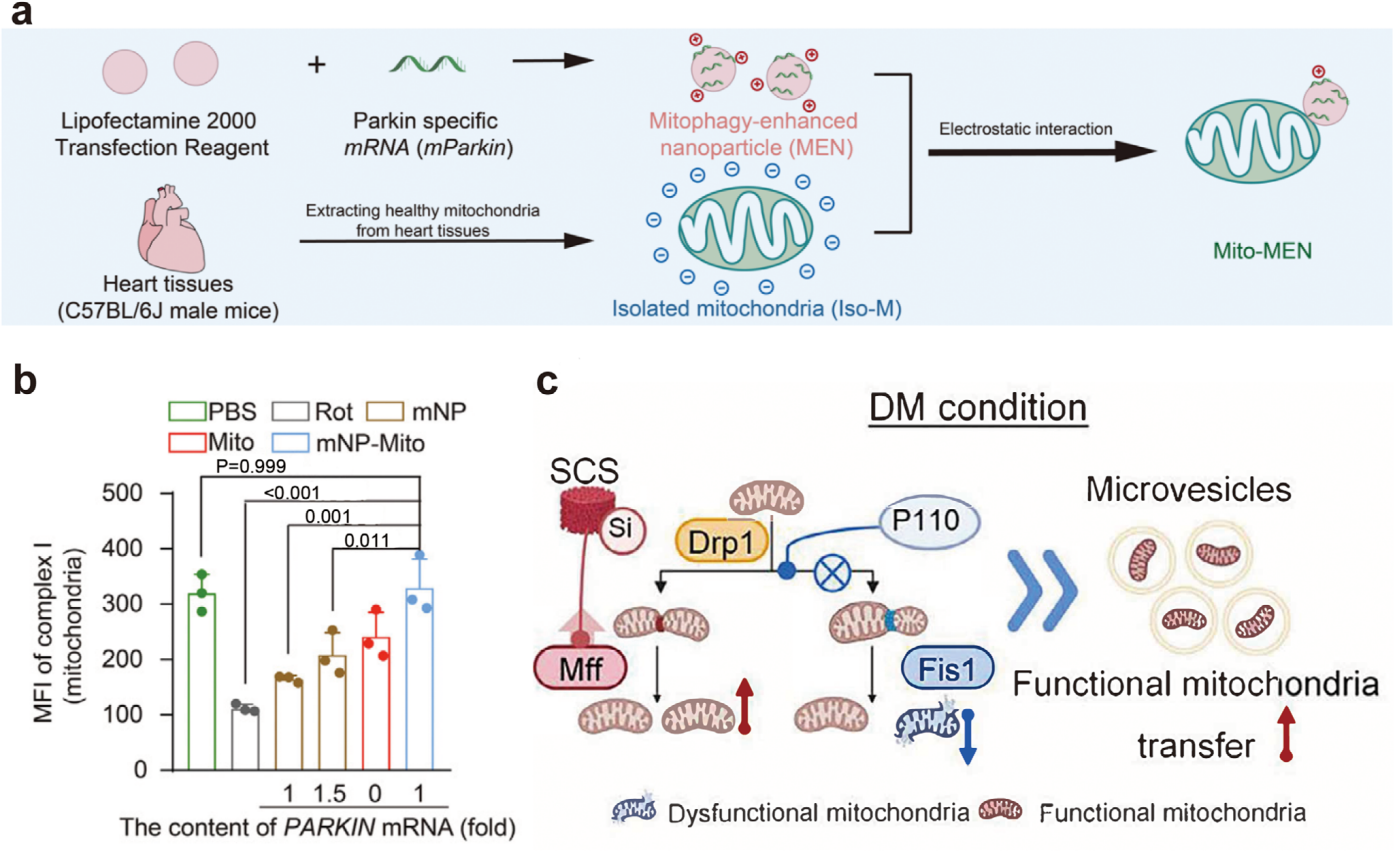
Integration of nanomaterials with mitochondrial quality control systems. (a) Construction of Mito‐MEN through the binding of mitophagy‐enhanced NPs to isolated mitochondria. Reproduced with permission [171]. Copyright 2024, American Chemical Society. (b) Detection of complex I content by flow cytometry in extracted mitochondria from different groups. Reproduced with permission [227]. Copyright 2024, Elsevier. (c) Combination of Si and P110 (inhibitor of the Drp1‐Fis1 interaction) alters mitochondrial fission to produce functional mitochondria. Reproduced with permission [226]. Copyright 2025, Wiley‐VCH GbmH.

Silicon enhances mitochondrial function in macrophages by modulating fission dynamics through the Drp1‐Mff pathway. This promotes the packaging of functional mitochondria into MVs and their subsequent transfer from macrophages to ECs and neuronal cells, supporting vascular and neural protection during early wound healing. Additionally, the inhibition of the Drp1‐Fis1 interaction fine‐tunes mitochondrial fission and augments the delivery of healthy mitochondria to stressed recipient cells (Figure 7c) [226].

#### 8.2.5 Engineering Donor Cells for High‐Quality Mitochondria

As a common mitochondria provider, MSC sources differ in their mitochondrial donation capacity. For instance, induced pluripotent stem cell (iPSC)‐MSCs outperform BM‐MSCs in mitigating anthracycline‐induced cardiomyopathy, owing to their intrinsic MIRO1 overexpression and enhanced TNF‐α‐responsive TNT formation, which collectively facilitate more efficient mitochondrial delivery and bioenergetic rescue [232]. Rapidly expanding clones (RECs), which were isolated as single clones from the CD90^high^/CD271^high^ population of bone marrow mononuclear cells, exhibit characteristics of highly purified MSCs. Following co‐culture with ρ0 cells, RECs exhibited a significant increase in the relative fluorescence intensity of CX43 and showed more dependence on mitochondrial transfer through GJs and MVs than MSCs. Compared to conventional MSCs, RECs demonstrated a greater capacity to transfer mitochondria to ρ0 cells [248]. Mitochondria isolated from the human osteosarcoma cell line MG63 exhibited a superior ability to direct MSCs toward an osteogenic fate compared to MSC‐derived mitochondria. This enhanced effect is characterized by the upregulation of key osteogenic markers (Runx2, osterix, and osteopontin) and activation of BMP2 signaling and calcium import, and it is mechanistically driven by the Wnt/β‐catenin pathway (Figure 8a,b). The therapeutic potential was confirmed *in v*ivo, where MSC spheroids loaded with MG63 mitochondria significantly enhanced bone regeneration in rodent defect models [228].

**FIGURE 8:**
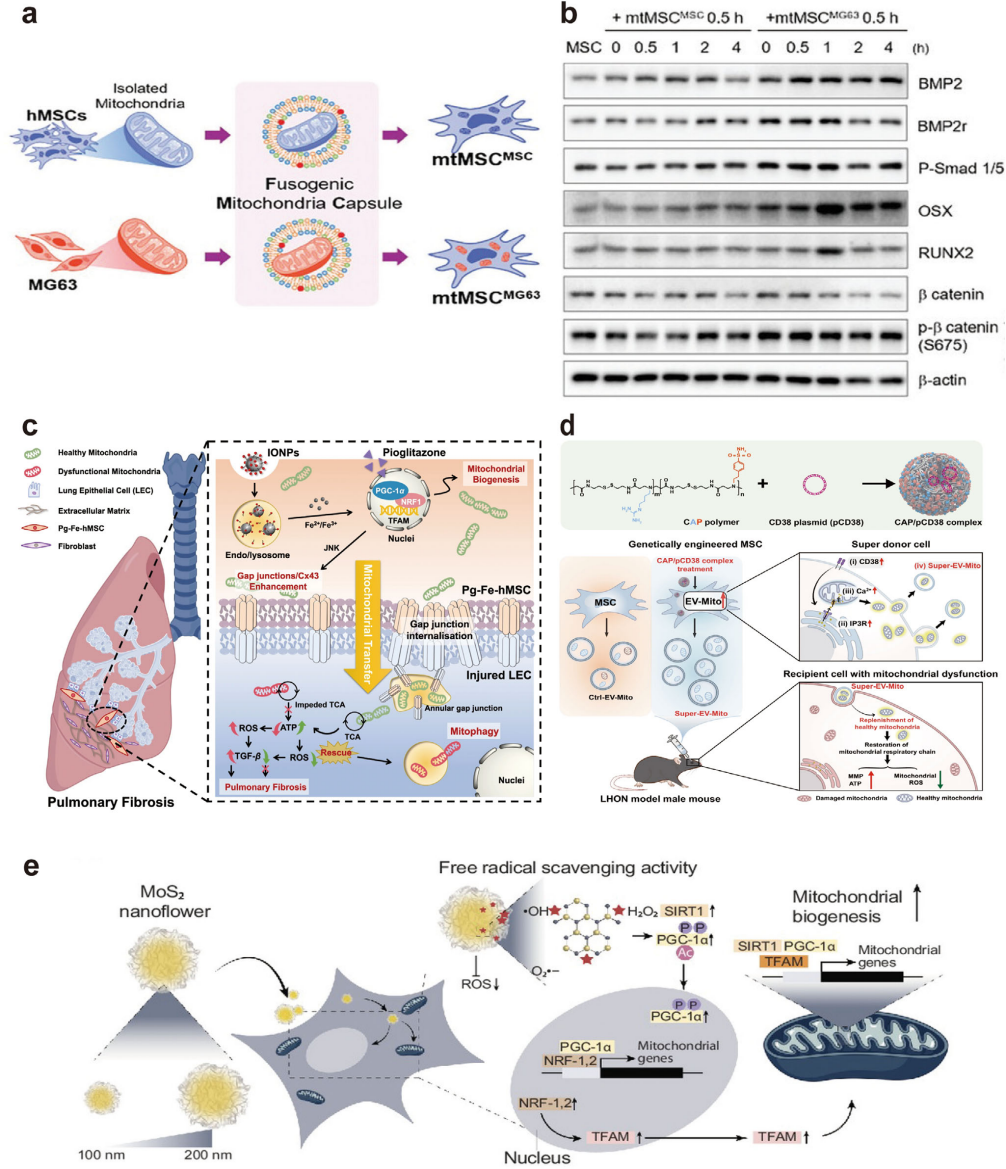
Nanomaterials engineer donor cells to yield high‐quality mitochondria. (a, b) Mitochondria isolated from MG63 exhibit a superior ability to direct mesenchymal stem cells (MSCs) toward an osteogenic fate through the activation of BMP2 signaling and the Wnt/β‐catenin signaling pathway. Reproduced with permission [228]. Copyright 2025, Wiley‐VCH GmbH. (c) Schematic illustration of Pg‐Fe‐hMSC for the treatment of pulmonary fibrosis. Reproduced with permission [27]. Copyright 2023, Springer Nature. (d) Schematic illustration shows the strategy of Super‐EV‐Mito to empower mitochondrial transfer. Reproduced with permission [28]. Copyright 2025, Springer Nature. (e) Schematic illustration of MoS_2_ nanoflower to scavenge reactive oxygen species (ROS) and trigger mitochondrial biogenesis. Reproduced with permission [229]. Copyright 2025, National Academy of Sciences.

Some studies incorporated additional components into nanomaterial systems to enhance the mitochondrial biogenesis capacity of donor cells. Huang et al. reported that pioglitazone (Pg), a clinical drug for diabetes, can activate mitochondrial biogenesis in human MSCs, primarily through the PGC1α‐NRF1‐TFAM pathway. Following treatment with Pg, IONPs were added to upregulate CX43 expression in MSCs, thereby establishing a synergistic engineering strategy that significantly increased mitochondrial transfer to injured lung cells (Figure 8c). This approach provided more efficient and prolonged mitochondrial transfer. Additionally, the enhanced mitochondrial delivery promoted the functional recovery of recipient cells. These engineered human MSCs (hMSCs) exhibited significant therapeutic efficacy in attenuating PF progression in a mouse model [27]. Wang et al. found that the CD38/IP3R/Ca^2+^ signaling axis regulates the release of mitochondrial‐containing EVs from MSCs. The nanomaterial CAP, comprising N, N’‐cystamine‐bis‐acrylamide (CBA), agmatine dihydrochloride (Agm), and 4‐(2‐aminoethyl) benzenesulfonamide (PAEBS), was used to form stable complexes with plasmid DNA encoding CD38. By augmenting the pathway through NP‐mediated gene delivery, the authors generated “super donor” MSCs. These engineered cells produced “Super‐EV‐Mito” at a yield 3‐fold higher than control EV‐Mito derived from unmodified MSCs (Figure 8d) [28].

Soukar et al. reported a biomaterial‐based strategy utilizing molybdenum disulfide (MoS_2_) nanoflowers with atomic‐scale vacancies, which confer the ability to scavenge ROS and stimulate the expression of genes associated with mitochondrial biogenesis. These nanoflowers successfully transform hMSCs into potent mitochondrial biofactories, doubling the mitochondrial mass within MSCs and significantly enhancing the intercellular transfer of mitochondria to recipient cells through TNTs (Figure 8e). This augmented transfer efficiently restores mitochondrial respiratory function and ATP production in recipient cells, demonstrating remarkable efficacy in rescuing cellular function in mitochondrial damage models [229].

#### 8.2.6 Prolonging Functional Persistence of Transplanted Mitochondria

Hydrogels are recognized as promising platforms because of their high‐water content, excellent biocompatibility, and tunable mechanical properties. They facilitate localized and sustained drug release and provide a supportive microenvironment for cell proliferation and tissue regeneration.

Microneedle arrays have attracted considerable interest as transdermal delivery platforms because of their capacity to enable precise and controllable drug administration into specific skin layers, together with advantages such as minimal invasiveness and high efficiency. Yao et al. fabricated a hydrogel microneedle patch (MNP) using decellularized adipose matrix and hyaluronic acid methacryloyl. They mixed this patch with metformin‐pretreated, mitochondria‐rich EVs derived from ADSCs (Figure 9a). Metformin acts as a mitochondrial biogenic agent, and its treatment increases mitochondrial area [249]. The MNP mechanically poked the wound biofilm layer and gradually released functional mitochondria into the local tissue. This process ameliorated mitochondrial dysfunction in the wound and promoted macrophage polarization toward the M2 phenotype. Ultimately, this strategy markedly enhanced wound healing in a mouse model of combined radiation and skin injury [30]. Similarly, Dong et al. used a soluble polyvinyl alcohol microneedle patch for the transdermal delivery of mitochondria encapsulated in EVs (Figure 9b) [25].

**FIGURE 9:**
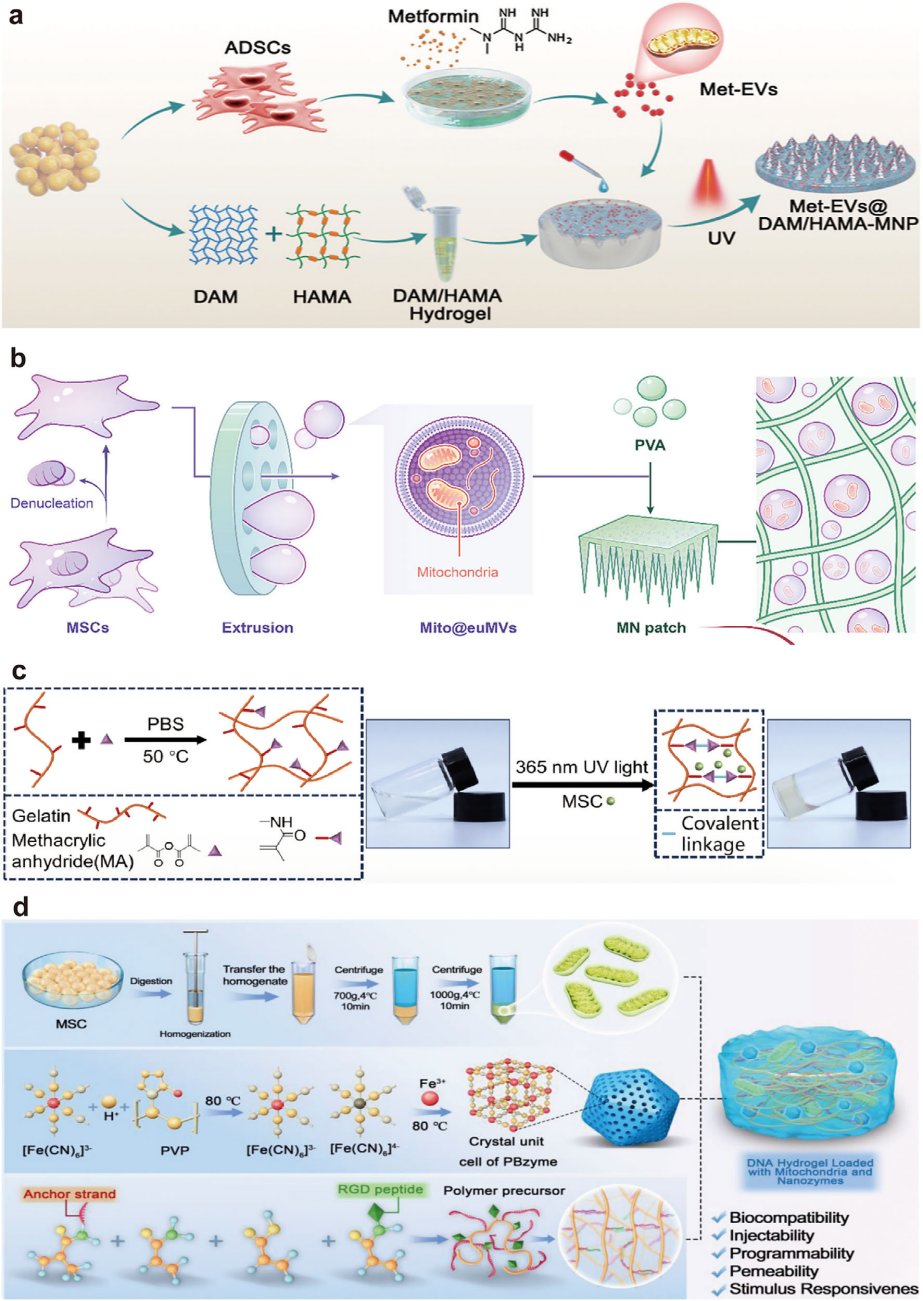
Utilization of nanomaterials to prolong functional persistence of transplanted mitochondria. (a) Preparation of the microneedle patch loaded with metformin‐pretreated, mitochondria‐rich EVs. Reproduced with permission [30]. Copyright 2024, American Chemical Society. (b) Preparation of the microneedle patch loaded with the enucleated mesenchymal stem cells (MSCs)‐derived microvesicles (MVs) containing mitochondria (Mito@euMVs). Reproduced with permission [25]. Copyright 2025, Wiley‐VCH GmbH. (c) Preparation of the methacrylate‐modified gelatin (GelMA) hydrogel for the sustained release of MSCs. Reproduced with permission [230]. Copyright 2025, Elsevier. (d) Preparation of the polymer‐DNA hybrid hydrogel loaded with mitochondria and reactive oxygen species (ROS) scavenging nanozymes. Reproduced with permission [221]. Copyright 2025, Elsevier.

To extend the therapeutic presence of MSCs after surgical brain injury, a methacrylate‐modified gelatin (GelMA) hydrogel was developed as a sustained‐release platform for MSCs (Figure 9c). The sustained release of MSCs from the biocompatible hydrogel facilitated mitochondrial transfer to host cells and provided significant therapeutic outcomes, including attenuated brain edema, reduced inflammation, and enhanced neurological function [230].

DNA hydrogels, fabricated through nucleic acid nanotechnology, offer distinct benefits such as programmable design and controllable degradation behavior. A novel polymer‐DNA hybrid hydrogel, functionalized with RGD peptides to promote cell adhesion, was developed to co‐deliver mitochondria and ROS‐scavenging nanozymes for RA treatment (Figure 9d). The hydrogel conferred immunocompatibility through polymer‐masked DNA sites and allowed degradation tuning to suit tissue repair kinetics. Combined with mitochondria and nanozyme delivery, it alleviated inflammation, enhanced chondrocyte function, and facilitated joint repair, as validated in experimental models [221].

#### 8.2.7 Preventing Lysosomal Degradation and Promoting Cytosolic Release

Because EVs and free mitochondria are usually taken up through endocytosis, their degradation by lysosomes is a major challenge. Previous studies have developed different strategies to avoid lysosomal degradation. Modifying appropriate molecules can help prevent lysosomal degradation. Pep‐1, a cell‐penetrating peptide, utilizes a noncovalent, self‐assembly mechanism to deliver cargo directly into cells. This endocytosis‐independent pathway allows the cargo to effectively bypass endolysosomal degradation [250]. As lysosomes are negatively charged, augmenting the positive surface charge of mitochondria‐targeting molecules such as TPP can promote lysosomal escape of nanozymes. TPP‐decorated nanomaterials have a stronger ability to escape lysosomal degradation than those without the TPP component [180].

According to the “proton sponge” hypothesis, PEI exhibits a high buffering capacity by binding to numerous protons within the lysosome. This promotes continuous proton influx through V‐ATPase, resulting in osmotic swelling and subsequent rupture of the organelle, thereby facilitating the escape [251, 252]. Based on their high density of carboxylate groups, PGA‐Cu‐S@G NPs facilitate endo/lysosomal escape primarily through a pH‐buffering mechanism. Following the acidification of endosomes, these carboxylate groups undergo dissociation (deprotonation), which locally buffers the decrease in pH. This buffering action consumes protons (H+) and generates a negative charge, thereby promoting a compensatory influx of additional protons and counterions into the endosomal lumen. The resulting ionic imbalance increases osmotic pressure, driving water influx and causing endosomal swelling and eventual rupture. Consequently, the NPs are released into the cytoplasm before fusion with lysosomes, effectively avoiding lysosomal degradation [166].

The MOF contains tunable molecular‐scale pores that permit essential ion and oxygen transport while effectively immobilizing and stabilizing the encapsulated cargo. The resulting porous barrier restricts the penetration of digestive enzymes, thereby protecting the cargo from biodegradation. Zhou et al. developed a strategy to encapsulate isolated mitochondria within a 1‐nm‐thick protective shell of zeolite imidazole framework‐8 (ZIF‐8). In addition to preserving mitochondrial bioactivity through physical encapsulation, the ZIF‐8 coating facilitates endosomal escape under acidic conditions through its degradation product, the imidazole ring, which promotes proton buffering and subsequent membrane disruption. The introduction of PEI provides a stronger ability to escape lysosomes. Equipped with the function of the cell‐penetrating peptide Tat, this dual mechanism ensures both protection and efficient intracellular delivery of functional mitochondria [220]. ZIF‐8 is also used for preparing the nanomaterial a127/mito@ZIF@Ma. Because of the coordination bonds between Zn^2+^ and 2‐methylimidazole, ZIF‐8 NPs exhibit pH‐sensitive biodegradability under acidic conditions. In a normal physiological environment (pH 7.4), anti‐miRNA‐127 shows a slow release profile, whereas its release is significantly accelerated in an acidic microenvironment (pH 5.5). The pH‐responsive properties of ZIF‐8 facilitate lysosomal escape and the release of anti‐miRNA‐127 and mitochondria. Based on this intrinsic targeting capability and pH‐responsive behavior of ZIF‐8, the nanocarrier enabled efficient and specific intracellular release of both mitochondria and anti‐inflammatory miRNA in macrophages. This combined strategy exerted a synergistic therapeutic effect by activating the innate immune response and protecting against bacteria‐ or virus‐induced ALI [29].

Some studies have designed systems that mimic routes that can bypass lysosomal degradation. Fusogenic mitochondrial capsules engineered by Kim et al. deliver their mitochondrial cargo directly into chondrocytes through membrane fusion, thus bypassing the endocytic pathway [228, 231]. Mitochondria transferred through the photothermal nanoblade can avoid lysosomal degradation as they are directly injected into recipient cells [216].

### 8.3 Biomimetic Nanomaterials as Artificial Mitochondria

Some studies used nanomaterials to directly mimic mitochondrial functions. Li et al. developed an orally administered artificial mitochondrial nanorobot (AMN), termed PFMACr, which was designed to directly supply ATP to damaged cells. This system comprises three functional units: a motion unit (M‐Arg) for chemotactic targeting to damaged mitochondria through iNOS recognition, an energy‐generating unit (phosphocreatine [PCr]) that provides high‐energy phosphate bonds to regenerate ATP from ADP in the cytoplasm, and a barrier‐crossing unit (PFA) that enables mucus penetration and intestinal absorption (Figure 10a). After reaching ischemic cardiac tissue, PFMACr AMNs not only rapidly produce ATP to alleviate mitochondrial energy deficits and restore cellular function but also scavenge ROS and generate anti‐inflammatory NO, which further supports mitochondrial recovery (Figure 10b). This multifunctional nanorobot is a promising oral strategy for treating energy deficiency diseases associated with mitochondrial dysfunction [32].

**FIGURE 10:**
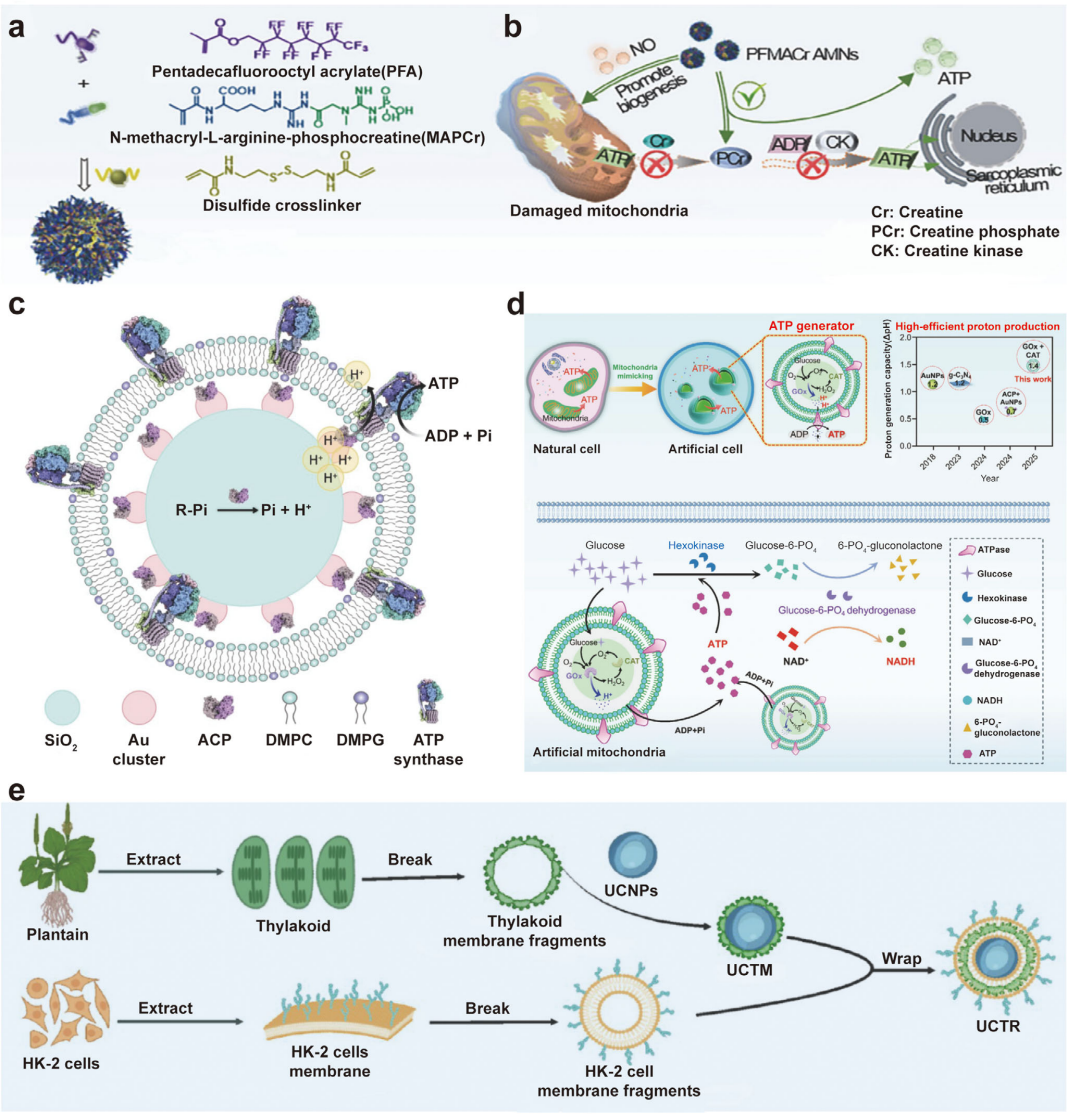
Biomimetic nanomaterials functioning as the artificial mitochondrion. (a) Process for preparing PFMACr AMNs. (b) PFMACr AMNs release NO to promote the biogenesis of damaged mitochondria and supply phosphocreatine (PCr) to enhance ATP production. Reproduced with permission [32]. Copyright 2025, Wiley‐VCH GmbH. (c) Construction of a supramolecular microreactor by reconstituting acid phosphatase (ACP) and ATP synthase with plasmonic Au clusters. Reproduced with permission [31]. Copyright 2024, Wiley‐VCH GmbH. (d) Construction of self‑energy‑supplying artificial cells and their application in NADH biosynthesis. Reproduced with permission [34]. Copyright 2025, Wiley‐VCH GmbH. (e) Process for preparing UCTR. Reproduced with permission [33]. Copyright 2025, American Chemical Society.

Xu et al. constructed a cell‐like supramolecular architecture by integrating acid phosphatase (ACP) and ATP synthase with plasmonic Au clusters (Figure 10c). This hybrid system utilizes the unique properties of Au clusters to achieve plasmon‐enhanced ATP synthesis: under light, the localized surface plasmon resonance effect generates photothermal heat that enhances ACP activity, thereby increasing the release of protons and inorganic phosphate (Pi). These products collectively promote ATP synthase for efficient phosphorylation. Simultaneously, the Au clusters confer robust resistance to abiotic stress by scavenging ROS to protect enzymatic activity and alleviating phosphorus deficiency through continuous Pi supply, which enables sustained energy production under challenging conditions [31].

Fu et al. fabricated a mitochondria‐mimicking ATP nanogenerator. This ATP generator comprises an inner mesoporous silica nanocapsule co‐loaded with glucose oxidase (GOx) and CAT and encapsulated within an outer ATPase‐integrated liposome bilayer. In the compartmentalized space, CAT decomposes H_2_O_2_ to generate O_2_, which is subsequently consumed by GOx to establish a self‐sustaining enzymatic cycle that amplifies proton production and creates a strong transmembrane proton gradient (pH shift from 7.37 to 3.77). The coating of these nanocapsules with an ATP synthase‐incorporated lipid bilayer produced the artificial mitochondrion. The resulting proton gradient across this membrane effectively influences the rotation of ATP synthase, enabling ATP generation. Following its integration into giant unilamellar vesicles as synthetic cells, this system supports autonomous NADH biosynthesis and glucose‐powered OXPHOS, mimicking the key metabolic features of living mitochondria (Figure 10d) [34].

Hu et al. engineered a bioenergetic nanoplatform (UCTR) regulated by second NIR (NIR‐II) light to address mitochondrial dysfunction in AKI. The platform is constructed using upconversion NPs (UCNPs) encapsulated within a thylakoid membrane (TM) and cloaked with membranes derived from activated renal tubular epithelial cells, which facilitates targeted delivery to injured tubules (Figure 10e). Following irradiation with deep tissue‐penetrating NIR‐II light, the UCNPs convert the NIR light into wavelengths of visible light to activate photosynthesis within the TM, thereby generating ATP and NADPH. This exogenous bioenergetic supply ameliorates hypoxia‐induced energy deficits and activates the AMPK/PGC‐1α pathway to restore MMP. UCTR treatment leverages the inherent antioxidative and anti‐inflammatory properties of plantain, thereby synergistically accelerating tubular repair [33].

## 9 Conclusion and Perspectives

Over the past decade, research on mitochondrial therapy has progressively deepened our understanding of its therapeutic potential. While therapeutic modulation of dysfunctional mitochondria, mitochondrial transplantation and mitochondrial transfer share the common goal of restoring mitochondrial homeostasis, they differ substantially in their mechanisms, advantages, and limitations.

Therapeutic modulation of dysfunctional mitochondria aims to repair or protect already damaged mitochondria through pharmacological agents. It does not require the introduction of exogenous mitochondria, thereby avoiding concerns regarding immunogenicity, source availability, and ethical considerations associated with mitochondrial procurement. Nanomaterials can be rationally designed to achieve precise targeting and deliver therapeutic payloads that effectively restore mitochondrial function. However, therapeutic efficacy is often limited by the extent of pre‐existing mitochondrial damage, as severely dysfunctional mitochondria may be beyond pharmacological repair.

Mitochondrial transplantation involves the direct delivery of exogenously isolated, healthy mitochondria to damaged tissues. It offers dose‐dependent therapeutic intervention and is particularly suited for acute injuries where rapid mitochondrial restoration is critical. However, uncertainties persist regarding optimal dosing, and whether metabolic differences between donor and recipient cells affect functional integration remains poorly understood. In addition, this approach faces well‐recognized challenges, including ex vivo preservation difficulties, allogeneic immune risks, and ethical concerns surrounding mitochondrial sourcing. Alternative sources, such as edible plant‐derived mitochondria, have been explored for their immune tolerance and pH stability [253], though their therapeutic efficacy requires further validation. Nanocarrier‐based strategies are primarily applied to facilitating targeted delivery, protecting mitochondria during preservation and prolonging their circulation time.

Intercellular mitochondrial transfer leverages the intrinsic capacity of cells to spontaneously donate and receive mitochondria. This physiologically compatible strategy minimizes immune rejection but is constrained by low transfer efficiency and incomplete mechanistic understanding. Meanwhile, within a pathological microenvironment, multiple cell types may engage in bidirectional or indiscriminate exchange, complicating targeted intervention. As commonly employed donor cells, MSCs have immunomodulatory properties and mitochondrial donation capacity [240, 254, 255], though their use introduces additional concerns such as the risk of oncogenic transformation and off‐target differentiation [256, 257]. This highlights the lack of safe and efficient donor cells. Hence, nanomaterials are being developed to optimize donor cell performance, construct artificial transfer channels or modulate endogenous pathways, and reshape the local microenvironment to improve transfer efficiency.

While many nanocarriers are designed to be biocompatible, certain nanomaterials, such as cobalt and silver nanoparticles, have demonstrated neurotoxic potential [258], and nanoplastics have been shown to promote senescence and cardiac aging through the cGAS–STING pathway [259, 260], underscoring the need for rigorous safety evaluation. Encouragingly, despite these concerns, a growing number of clinical trials on mitochondrial transplantation have been initiated across diverse disease settings, including myocardial infarction, acute ischemic stroke, and inflammatory myopathy. Therapeutic modulation of dysfunctional mitochondria has also been clinically explored. These ongoing efforts, together with advancing nanomaterial technologies, could accelerate the eventual translation of mitochondrial therapies.

Several other translational challenges must be addressed. The absence of a clear regulatory framework creates substantial uncertainty for clinical development. Importantly, the long‐term risks of exogenous mitochondrial transplantation, such as unpredictable integration into host cells, and the possibility of unintended genetic or epigenetic alterations, require thorough evaluation and dedicated regulatory oversight. Scalable manufacturing is also challenged by process stability and batch‐to‐batch consistency for both mitochondrial and nanocarrier production.

Looking forward, a deeper understanding of the molecular determinants of mitochondrial transfer specificity and efficiency is urgently needed to inform the rational design of advanced nanocarriers. Ideal nanocarrier designs should have precise targeting ability, stimuli‐responsive release, immune‐evasive surface modifications, and favorable safety and stability profiles. Importantly, while most current studies rely on conventional cell cultures or animal models, validation in organoid systems would provide more physiologically relevant efficacy and safety assessments. The convergence of mitochondrial therapies with AI‐assisted formulation design and real‐time in vivo imaging might open new frontiers for personalized mitochondrial medicine.

In summary, mitochondrial therapy, empowered by nanomaterial engineering, represents a promising therapeutic paradigm for diseases rooted in mitochondrial dysfunction. Successful clinical translation will require continued innovation in both material science and mitochondrial biology, alongside robust regulatory frameworks, scalable manufacturing solutions, and comprehensive long‐term safety evaluation. With sustained interdisciplinary effort, nanoengineered mitochondrial therapies hold the potential to fundamentally reshape the treatment landscape for patients with currently intractable mitochondrial dysfunction‐associated diseases.

## Author Contributions

J.D. and X.H.L. contributed key ideas that shaped the manuscript and provided supervision and strategic direction. Y.Y.S. draft and refined the manuscript. Y.Y.S., Z.Y.G., and C.L.Z. designed and organized the figures and tables. J.M., L.L.H., P.F.Y., L.S.Z., and L.L.Y. revised and refined the manuscript. All authors have read and approved the review article.

## Conflicts of Interest

The authors declare no conflicts of interest.

## Data Availability

Data sharing not applicable to this article, as no datasets were generated or analysed during the current study.
